# Diabetes in spotlight: current knowledge and perspectives of photobiomodulation utilization

**DOI:** 10.3389/fendo.2024.1303638

**Published:** 2024-03-19

**Authors:** Quentin Perrier, Cécile Moro, Sandrine Lablanche

**Affiliations:** ^1^ Univ. Grenoble Alpes, INSERM U1055, Pharmacy Department, Grenoble Alpes University Hospital, Laboratory of Fundamental and Applied Bioenergetics, Grenoble, France; ^2^ Univ. Grenoble Alpes, CEA-Leti, Clinatec, Grenoble, France; ^3^ Univ. Grenoble Alpes, INSERM U1055, Diabetology and Endocrinology Department, Grenoble Alpes University Hospital, Laboratory of Fundamental and Applied Bioenergetics, Grenoble, France

**Keywords:** photobiomodulation, diabetes, neuropathy, wound healing, periodontitis, retinopathy, glycemic control

## Abstract

**Introduction:**

Diabetes is a global health concern characterized by chronic hyperglycemia resulting from insulinopenia and/or insulin resistance. The rising prevalence of diabetes and its associated complications (ulcers, periodontitis, healing of bone defect, neuropathy, retinopathy, cardiopathy and nephropathy) necessitate innovative therapeutic approaches. Photobiomodulation (PBM), involves exposing tissues and cells to low-energy light radiation, leading to biological effects, largely via mitochondrial activation.

**Methods:**

This review evaluates preclinical and clinical studies exploring the potential of PBM in diabetes and its complications, as well all clinical trials, both planned and completed, available on ClinicalTrials database.

**Results:**

This review highlights the variability in PBM parameters across studies, hindering consensus on optimal protocols. Standardization of treatment parameters and rigorous clinical trials are needed to unlock PBM’s full therapeutic potential. 87 clinical trials were identified that investigated PBM in diabetes mellitus (with 5,837 patients planned to be treated with PBM). Clinical trials assessing PBM effects on diabetic neuropathy revealed pain reduction and potential quality of life improvement. Studies focusing on wound healing indicated encouraging results, with PBM enhancing angiogenesis, fibroblast proliferation, and collagen density. PBM’s impact on diabetic retinopathy remains inconclusive however, requiring further investigation. In glycemic control, PBM exhibits positive effects on metabolic parameters, including glucose tolerance and insulin resistance.

**Conclusion:**

Clinical studies have reported PBM-induced reductions in fasting and postprandial glycemia without an increased hypoglycemic risk. This impact of PBM may be related to its effects on the beta cells and islets in the pancreas. Notwithstanding challenges, PBM emerges as a promising adjunctive therapy for managing diabetic neuropathy, wound healing, and glycemic control. Further investigation into its impact on diabetic retinopathy and muscle recovery is warranted.

## 1 Introduction

Diabetes is characterized by chronic hyperglycemia due to insulinopenia [type 1 diabetes (T1D)] and/or insulin resistance [type 2 diabetes (T2D)]. The International Diabetes Federation reported 537 million of potential cases of diabetes across the world in 2021 with an increment planned for 2045 at 783 million of potential cases (1). As a result, diabetes caused 6.7 million of death in 2021 (1) and led to USD 966 billion health expenditures (1) partially due to numerous complications related to diabetes disease (2) such as macrovascular complication (ischemic cardiomyopathy, stroke and arteriopathy) (2–5) and microvascular complications: 1) retinopathy, diabetes is the first cause of non-traumatic blindness (6), 2) nephropathy, diabetes is the first cause of dialysis, 3) amputation and 4) neuropathy (7, 8) leading to foot ulceration and exposing patient to a risk of lower limb amputation [diabetes is the first cause of non-traumatic amputation (9)]. Finally, patients living with diabetes are also exposed to a risk of periodontitis (10). Altogether, diabetic complications alter quality of life (11, 12). These complications can be prevented through optimal glycemic control and could be managed with some medicines (13, 14). However, despite optimal medical management, prevention of diabetic complications remains a challenge and additional treatment remains mandatory.

Light was used as potential of treatment since the ancient Egypt. The biological reaction to light and its therapeutic applications are not new. For example, the beneficial effect of light on neonatal jaundice, discovered in the 1950s, made phototherapy (with blue light with a wavelength between 420-490 nm) the main modality for its treatment (15). Another example, the effects of light on mood, demonstrated in the 80s, made it possible to propose light therapy as a treatment for seasonal affective disorders, and it has recently been shown to have an effect comparable to antidepressants in episodes major depression (16).

Photobiomodulation therapy (PBM), formerly called “Low level laser therapy”, is a phototherapy based on the exposure of tissues and cells to non-ionizing and very weak light radiation with a wavelength generally ranging from red (between 600-700 nm) to the near infrared (between 700-1400 nm) and resulting in biological effects following its absorption by endogenous chromophores. Historically, PBM was first described by Endre Mester in 1968 who observed faster hair regrowth in rodents exposed to a low-energy laser with a wavelength of 694 nm (17, 18). In the same years, it was also developed by the National Aeronautics and Spatial Administration (NASA) to accelerate the healing and regeneration of muscle cells in astronauts (19). For the past decades, biomedical research relating to PBM has been constantly increasing, indicating a growing interest in its therapeutic potential. Interest in PBM has also been linked to technical developments in illumination technology, with the improvement of LEDs, which are cheaper, safer and give off less heat than lasers (20). Depending on the targeted use, and the illumination device, PBM can be brought along white light, to have the full spectrum of wavelength as a natural light, or along LED to obtain mainly a light targeted around a wavelength, or a laser to deliver only a define wavelength. At the beginning of the 2000s, the use of PBM in aesthetics (hair regrowth, wrinkle reduction) and sports recovery helped to democratize its use. Photobiomodulation is now used to help heal damaged tissues, improve immune response, reduce inflammation, and was recommended to prevent or treat certain side effects of treatments such as chemotherapy and radiotherapy (mucositis and radiodermatitis). In 2010, the first clinical authorization was reached as therapeutics for pain in conditions such as osteoarthritis. Since the 2020s, based on successful preclinical researches, various clinical trials have been initiated to evaluate PBM as a treatment to slow down neurodegenerative diseases, such as Alzheimer’s or Parkinson’s diseases (21, 22). Over the past three years (2020–2022), approximately 850 articles per year have been published and referenced in MEDLINE.

The main mechanism of action involves the mitochondria (23), possessing photo-acceptors sensitive to the lengths used with PBM. Briefly, PBM has been reported to activate non-mitochondrial cellular functions (light/heat-gated ion channels) and restore mitochondrial function (through interfacial water and/or activation of cytochrome C oxidase), resulting in a short-term increase adenosine triphosphate (ATP) energy production in body cells and increased production of NO. This process leads to long-term effects, with the expression of various stimulatory and protective genes. The main biological effects highlighted in preclinical and clinical studies are an anti-inflammatory, analgesic action, an increase in blood circulation, angiogenesis, and a healing/regeneration and tissue proliferation action (24–26). Given these effects, the potential therapeutic applications are numerous. PBM is already used in certain medical disciplines. It is part, for example, of the recommendations for the prevention of mucositis in patients treated by radiotherapy.

The use of PBM in the context of neurodegenerative diseases, and in particular Parkinson’s disease, is currently being studied and is the subject of clinical trials. PBM could represent an innovative therapeutic solution, to slow down the neurodegenerative process. The preclinical results in this direction are very encouraging, and clinical data should be published soon due to ongoing clinical trials. The metabolic syndrome in neurodegenerative diseases, and in particular Parkinson’s disease, are well established (21, 22). The observed effect of PBM on cellular metabolism, inflammatory and scarring processes is a lead that may indicate an interest of PBM in the regulation of phenomena related to metabolic syndromes, such as diabetes.

In the present work, the purpose is to review the clinical studies using PBM and conducted in the field of the treatment of diabetes and diabetic complications.

## 2 Materials and methods

Identification was made regarding:

- Pre-clinical (animal study) and clinical data available until 23 May 2023
- Clinical trials available on clinicaltrials.gov (27) until 23 May 2023

### 2.1 Searching strategy and selection criteria of papers

A MEDLINE research was conducted via PubMed using the search terms: [(Photobiomodulation) or (Low-level laser therapy) or (Near-infrared therapy)] AND (diabetes or diabetic or T1D or T2D) AND (1900/01/01:2023/05/23[edat]). In addition, references from cited papers were investigated. For each paper, the following parameters were recorded: 1st author, year of publication, model used (including the number of patients in clinical trials), wavelength, light source (LED or laser), mode of administration (continuous or pulsed), PBM parameters (power density in mW/cm², time per exposure, energy density in J/cm², frequency, sites), and the study’s conclusion. The data were analyzed both collectively and individually, considering different diabetes conditions such as retinopathy, ulcers, and periodontitis. In the case of clinical trial papers, it was specified whether they were randomized clinical trials (RCTs), pre-post interventional trials (Pre-Post ITV) or observational studies.

### 2.2 Searching strategy for clinical trials and classification

**Figure 1:**
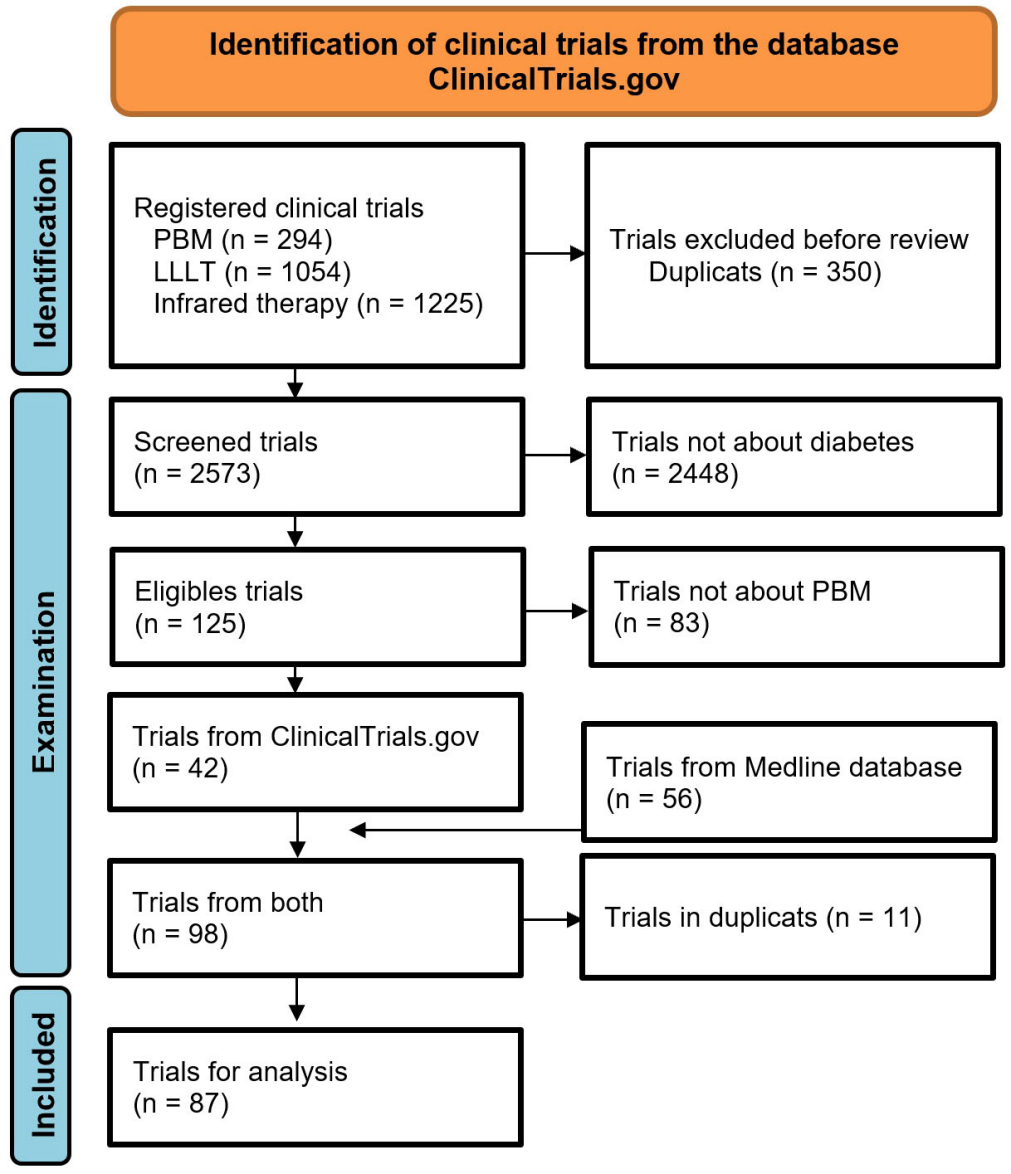
Flow chart for selection of clinical trials. PBM: photobiomodulation.

The screening of the ClinicalTrials database (27) was conducted using three terms: photobiomodulation, low-level laser therapy and near-infrared therapy. Only trials related to diabetes were considered eligible, and studies employing methods other than PBM were excluded (**Figure 1**). For each included trial, the following parameters were recorded: starting date, country of the sponsor, expected number of enrolled patients, and enrolment status (not yet recruiting, recruiting, enrolling by invitation, active, suspended, terminated, completed, withdrawn, or unknown). Regarding clinical trials published in the Medline database, the following parameters were recorded: country of the sponsor and number of enrolled patients. All these trials were classified as completed.

## 3 Results

**Figure 2:**
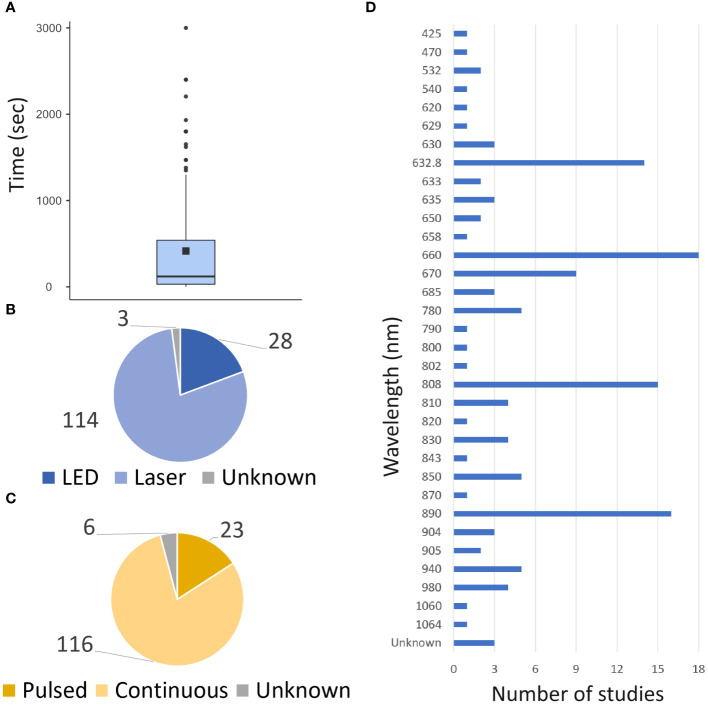
Global PBM parameters. The data from both preclinical and clinical studies are presented. **(A)** Repartition of time exposure to PBM. **(B)** Repartition of source of light uses. **(C)** repartition of the mode of illumination uses. **(D)** Repartition of wavelength uses.

Following Medline research, 124 articles met the search criteria. 170 different exposure times were studied and varied widely, with 35% of the studies (60/168) having exposure times (for one session) of less than a minute and 35% (59/168) exceeding 5 min (**Figure 2**). Laser was the predominant light source investigated in 80% of the studies (**Figure 2**), and continuous exposure was the primary mode of administration (84% of studies, **Figure 2**). The power density values ranged from 1 mW/cm² to 8.32 W/cm², whereas the energy density ranged from 0.03 to 420 J/cm². These studies investigated various wavelengths ranging from 425 to 1064 nm, with 82% of studies between 600 and 900 nm, and six studies exploring multiple wavelengths applying simultaneously (**Figure 2**). It should be noted that these parameters were not fully described or available in 37 studies (29%).

### 3.1 Clinical trials in humans

**Figure 3:**
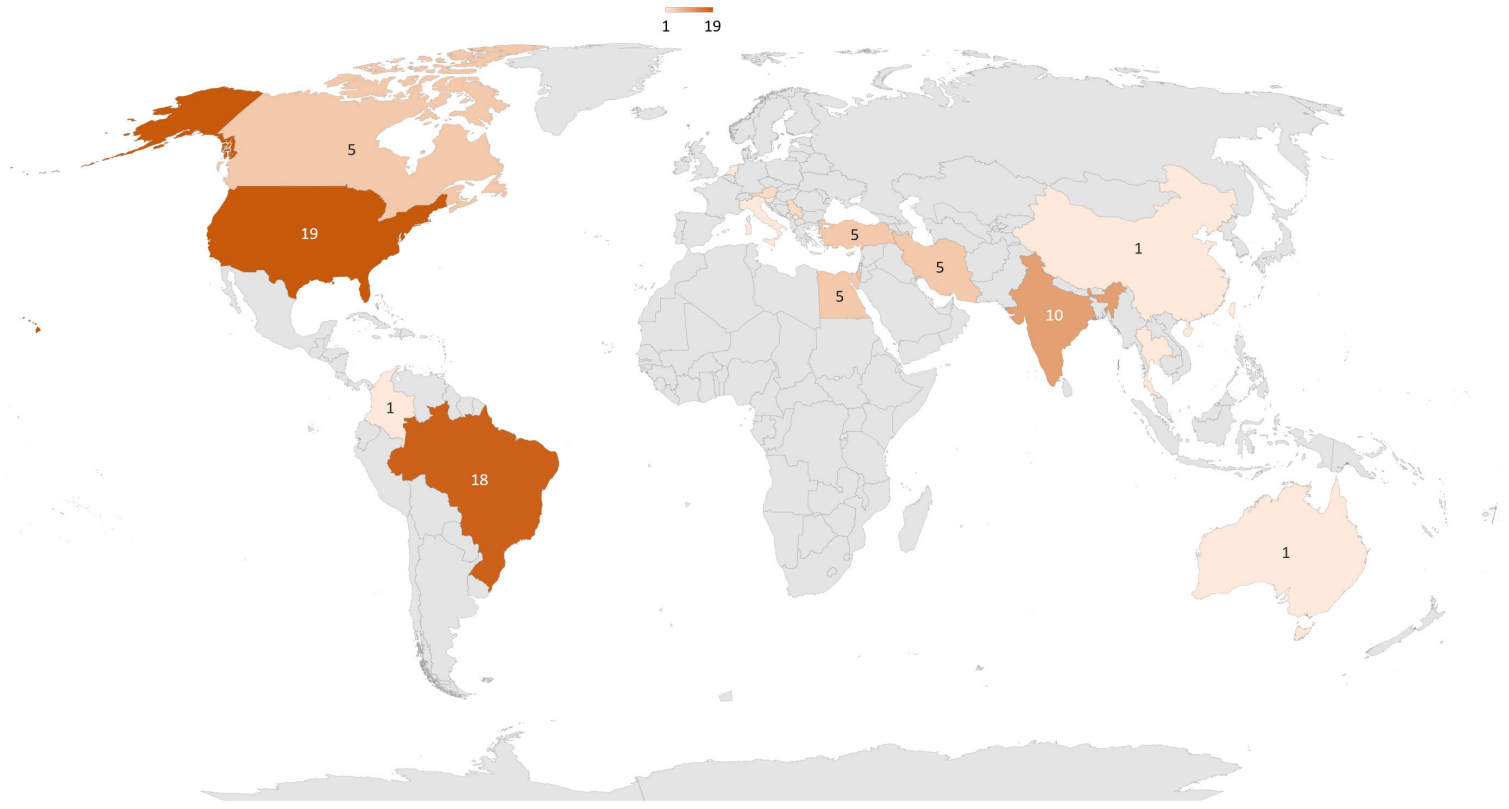
Repartition around the world of clinical trials about PBM and diabetes.87 trials have been conducted or are planned, 43 in America, 26 in Asia, 12 in Europe, 5 in Africa, and 1 in Oceania.

42 trials were identified in ClinicalTrial database (27), and 56 articles were found in Medline. After removing duplicate trials, 87 were conducted or planned (**Figure 2**). These trials included 5,837 patients (see **Table 1** for details). The top three countries planning to conduct trials on PBM and diabetes were the United States (n = 19), Brazil (n = 18), and India (n = 10, **Figure 3**). 50% of publications were by completely independent teams (n = 21 articles). Regarding diabetic neuropathies, two teams each published three studies: Burke et al. (28–30), and Arun G et al. (31–33). Regarding diabetes chronic periodontitis, three teams each published 2-3 studies: Chava et al. (34, 35), Haaki et al. (36, 37), and Obradovic et al. (38–40). Regarding performance and functionality during or after exercise in patient with diabetes Ferraresi et al. (41–43). published three studies. Finally, two teams have published on neuropathic pain and diabetic ulcers: Arisawa et al. (44, 45), and Schindl et al. (46–48).

**Table 1:** Number of patients treated in clinical trials with PBM for diabetes condition.

Status of studies	Number of studies	Expected number of participants
Active, not recruiting	1	80
Completed	66	4954
Not yet recruiting	2	160
Recruiting	5	300
Suspended	1	60
Terminated	4	75
Unknown status	6	208
Withdrawn	2	0
**Total**	**87**	**5837**

These numbers of participant represent the expected enrolment for trial not already completed.

### 3.2 Impact of PBM on diabetic neuropathy

A total of 23 studies were conducted, including 5 on preclinical models- (49–53) and 18 in humans (28–33, 46, 54–63) (**Table 2**).

**Table 2:** Effects of PBM on diabetic neuropathy.

Author/Year		Model	Wave (nm)	Light	PBM parameters	Conclusions
Vieira 2022 (49)		Lewis rat Streptozotocin induced	904	Continuous laser	7W/cm², 29sec, 203J/cm² Frequency: once a day Site: 1, dorsal, direct contact	↓ pain ↓ cytokines (TNFα, IL-1β, IL-10) No effect on metabolic parameters
Rocha 2021 (50)		Wistar rat Streptozotocin induced	904	Continuous laser	340mW/cm², 18sec, 6,23J/cm² Frequency: once a day, for 10 days Site: on sciatique nerve routes, direct contact	Restore fusion/fission mitochondria dynamic
Vieira 2019 (51)		Lewis rat Streptozotocin induced	904	Continuous laser	7W/cm², 29sec, 203J/cm² Frequency: once a day for 8 days Sites: 4 points, direct contact	↓ pain
Abdel-Wahhab 2018 (52)		Albinos rat Streptozotocin induced	808	Continuous laser	30sec, 90J* Frequency: 3 times a week for 8 weeks Sites: 3 points	↓ pain ↓ PGE2, TNFα, IL1β, IL10
da Silva Oliveira 2018 (53)		C57BL6 mice Streptozotocin induced	660	Continuous laser	107mW/cm², 15sec, 1.6J/cm² Frequency: once a day, for 21 days Site: 1, plantar hind paw, direct contact	↓ pain Prevent myelin degenerescence ↓ loss of C fiber
Rastogi 2021 (54)		Human, n = 38 Diabetes RCT^T^	890	LED Unknown	30min, 58.5J/cm²/min Frequency: 3 times a week, for 12 weeks Sites: 4, plantar foot x2, posterior & anterior distal leg	↓ pain (VAS decrease of 5.3 vs. 3 at 3 months) ↑ QoL (Norfolk-QoL DN 8 vs. 12 at 3 months) No effect on cutaneous reinnervation
Anju 2020 (31)		Human, n = 50 T2D Pre-Post ITV^ø^	632.8	Continuous laser	5.7mW/cm², 9min, 3.1J/cm² Frequency: once a day, for 10 days Sites: 2, plantar and dorsal foot	↓ vibration perception threshold ↓ neurone specific enolase
da Silva Leal 2020 (44)		Human, n = 30 T2D RCT^†^	660	Continuous laser	1.66mW/cm², 30min, 3J/cm² Frequency: once a day, for 10 days, 20 days washout and start 2 times more Site: 1, radial artery region, direct contact	↓ pain VAS: no change vs. decrease of 3 points LANSS: no change vs. decrease of 3 points Pain detect questionnaire: no change vs. decrease of 5 points ↑ QoL (SF-36, all parameters increase)
Anju 2019 (32)		Human, n = 40 T2D Pre-Post ITV^ø^	632.8	Continuous laser	5.7mW/cm², 9min, 3.1J/cm² Frequency: once a day, for 10 days Sites: 2, plantar and dorsal foot	↑ Mg and Vitamin D
Kumar 2015 (55)		Human, n = 19 T2D Pre-Post ITV^ø^	660 + 850	Continuous laser	5.7mW/cm², 9min, 3.1J/cm² Frequency: once a day, for 10 days Sites: 2, plantar and dorsal foot, direct contact	↓ pain (VAS: decrease of 5.2 points after 10 days) ↓vibration perception threshold ↑ microcirculation
Bashiri 2013 (56)		Human, n = 60 T1D & T2D RCT^†^	780	Continuous laser	8.3mW/cm², 5min, 2.5J/cm² Frequency: 2 times a week, for 4 weeks Site: unknown	↓ pain (VAS: score of 7.9 vs. 5.9 for PBM after 4 weeks)
Yamany 2012 (33)		Human, n = 30 T2D RCT^†^	850	Continuous laser	6.3mW/cm², 15min, 5.7J/cm² Frequency: 3 times a week, for 4 weeks Sites: 2, plantar surface of foot, lombo-sacral area, 30cm above the area	↓ pain (VAS; score of 6.9 vs. 5.3 for PBM after 4 weeks) ↑ microcirculation ↑ sural nerve conduction No effect on peroneal nerve
Khamesh 2011 (57)		Human, n = 27 T2D Pre-Post ITV^†^	800	Continuous laser	1W* Frequency: 10 sessions Sites: 6 paravertebral points, 4 on sciatic nerve routes	↑ neuronal potential amplitude
			905	Pulsed laser	25W*, 10J/cm² Frequency: 10 sessions Sites: 6 paravertebral points and 4 on sciatic nerve routes	↑ neuronal potential amplitude
Swisloki 2010 (58)		Human, n = 121 Diabetes RCT^†^	870	Continuous laser	350W*, 7min, 1800J* Frequency: Once a day, 4 days Sites: 16 on each foot	Restauration sensation No effect on pain (VAS, PQAS) No effect on quality of life (SF-36)
Lavery 2008 (59)		Human, n = 60 Diabetes RCT^ø^	890	Continuous LED	40min, 1,3J/cm²/min Frequency: once a day, for 90 days Sites: 4, plantar foot x2, medial & lateral side of the calf	No effect on pain (VAS) No effect on QoL (Neuro QoL) No effect on peripheral sensation
Arnall 2006 (60)		Human, n = 22 T1D & T2D RCT (for foot)^†^	880 + 650	Pulsed LED	30min Frequency: 3 times a week, for 8 weeks Sites: 2, volar and dorsum of foot	↑ mechanical sensation ↓ perception threshold ↑ peripheral protective sensation
Harkless 2006 (28)		Human, n = 979 Diabetes Pre-Post ITV^ø^	890	Pulsed laser	Unknown	↓ pain (VAS: decrease of 4.8 points) ↑ foot sensation
Clifft 2005 (61)		Human, n = 70 Diabetes RCT^ø^	890	Continuous laser	30min, 58.5J/cm²/min Frequency: 3 times a week, for 4 weeks Sites: 4, plantar foot x2, posterior & anterior distal leg	No effect on sensation
DeLellis 2005 (29)		Human, n = 790 T2D Pre-Post ITV^ø^	890	Pulsed laser	No detail	↓ sensitivity impairment Restauration protective sensation
Leonard 2004 (62)		Human, n = 27 T1D & T2D Pre-Post ITV^ø^	890	Continuous laser	40min, 50J/cm² Frequency: 3 times a week, for 4 weeks Sites: 4, inferior member	↓ pain (VAS: decrease of 2.3 points after 4 weeks) ↓place without sensation Restauration protective sensation
Zinman 2004 (63)		Human, n = 50 Diabetes RCT^†^	905	Continuous laser	60mW*, 5min Frequency: 2 times a week, for 4 weeks Sites: 2 painful sites, direct contact	No effect on pain (p = 0.07) (VAS)
Kochman 2002 (30)		Human, n = 49 T1D & T2D Pre-Post ITV^†^	Unknown	Continuous laser	30min Sites: 4, Posterior & anterior tibia, dorsal & ventral foot	↓ sensitive impairment ↑ neural function (hot/cold discrimination)
Schindl 2002 (46)		Human, n = 30 Diabetes RCT^ø^	632.8	Continuous laser	30J/cm² Frequency: Once Site: 1	↑ microcirculation

T: no other treatment for pain, ^†^:treatments for pain are permitted, but must be balanced and not change during the course of the study (i.e. amitryptilin, gabapentin, tramadol), ^ø^: no information provided about other treatment during the study, IL, Interleukin; LANSS, Leeds assessment of neuropathy symptoms and signs; LED, Light emitting diode; PBM, photobiomodulation; PQAS, Pain qualities assessment scale; Pre-Post ITV, Pre-post interventional trial; QoL, Quality of life; RCT, randomized controlled trial; T1D, Type 1 diabetes; T2D, Type 2 diabetes; TNF, Tumor necrosis factor; VAS, Visual analogic scale. *Surface of PBM not reported.↑, increse; ↓, decrease.

#### 3.2.1 Pain investigation

Among 14 studies investigating pain, 11 reported a decrease in pain after PBM, with 4 studies conducted on preclinical models and 7 on clinical subjects. In clinical trials, pain was evaluated using a visual analogic scale (VAS), either alone or in combination with other pain assessment scores with a follow-up period ranging from 4 to 90 days. For pre-post interventional trials (n = 3), the VAS scores decreased by an average of 2.3 to 5.2 points after PBM. In RCTs (n = 4), the VAS scores decreased more in the PBM group than in the control group (0 to 3 points *vs.* 3 to 5 points after 10 days) (44, 54), and the VAS scores at the end of the follow-up period were lower in the PBM group than in the control group (6.9-7.9 *vs.* 5.9-5.3) (33, 56).

#### 3.2.2 QoL investigation

Four studies investigated the effect of PBM on QoL. Two studies reported a positive impact of PBM on QoL associated with a decrease in pain (44, 54). Conversely, 2 studies did not report an impact of PBM on QoL but in these 2 studies PBM did not induced a decrease in pain (58, 59). The first study (58) used the highest power (350W), and the second study (59) used the longest exposure time (40 min) and application frequency (once a day for 90 days).

### 3.3 Impact of PBM on diabetic retinopathy

A total of 6 studies were conducted, including 4 studies on preclinical models (64–67) and 2 studies in humans (68, 69) (**Table 3**). Preclinical studies consistently reported a positive effect of PBM on retinal structure associated with a decrease in oxidative stress. In clinical studies focusing on macular oedema, findings were divergent regarding improvement of visual acuity between the two identified studies (68, 69). However, no side effects were reported in either study.

**Table 3:** Effect of PBM on diabetic retinopathy.

Author/Year	Model	Wave (nm)	Light	PBM parameters	Conclusions
Ahmed 2021 (64)	Wistar rat Streptozotocin induced	670	Continuous laser	5mW/cm², 90sec, 0.9J/cm² Frequency: 2 times a week, for 6 weeks Sites: 2, each eye, directly in eye	Improve structure of inner nuclear layer and retinal pigmented epithelium ↓ anatomical abnormalities
Cheng 2018 (65)	C57BL/6J mice Streptozotocin induced	670	Continuous LED	25mW/cm², 240sec, 6J/cm² Frequency: once a day, for 8 months Site: 1, back of the animal	↓ degenerescence of retinal capillaries ↓ albumin accumulation in the inner nuclear and in the outer plexiform layers of the retina Preservation of spatial frequency threshold contrast sensitivity
Saliba 2015 (66)	C57BL/6J mice Streptozotocin induced	670	Continuous LED	20mW/cm², 240sec, 5J/cm² Frequency: once a day, for 10 weeks Site: Total body	↓ superoxyde in retina ↓ abnormalities induced in leukostasis No effect on contrast sensitivity
Tang 2013 (67)	Lewis rat Streptozotocin induced	670	Continuous LED	25mW/cm², 240sec, 6J/cm² Frequency: once a day, for 10 weeks Site: total body	↓ diabetes induced abnormality of retinal function and abnormalities of electroretinograms ↓ retinal ganglion cell death Inhibited leukostasis
Shen 2020 (68)	Human, n = 21 Diabetes Pre-Post ITV^†^	670	Continuous LED	25-100-200mW/cm², 90sec, 2.25-9-18J/cm² Frequency: 12 sessions over 5 weeks Site: fundus contact lens	↓ central macular thickness (100 and 200>25) No effect on visual acuity No adverse event
Eells 2017 (69)	Human, n = 10 Diabetes RCT^†^	670	Unknown	45mW/cm², 100sec, 4.5J/cm² Frequency: 3 consecutive days per week, for 8 weeks Sites: 2, each eye, directly in eye	↓ central retinal thickness ↑ visual acuity (+ 6 letters)

^†^: PBM in addition to the best standard of care, at the investigator discretion choice, LED, Light emitting diode; Pre-post ITV, Pre-post interventional trial; RCT, Randomized controlled trial.↑, increse; ↓, decrease.

### 3.4 Impact of PBM on glucose metabolism in T2D

A total of 9 studies were conducted, with 8 studies conducted on preclinical models (70–78) and one study in humans (79) (**Table 4**). The preclinical studies consistently reported positive effects of PBM on metabolic parameters with an improvement in glucose tolerance [with a glucose tolerance test area under the curve (GTT AUC) decreasing by 12–28%], a decrease in insulin resistance [with a 22% decrease in homeostatic model assessment of insulin resistance (HOMA-IR (70)) and a 10% decrease in insulin tolerance test (ITT) AUC (72)] and a decrease in fasting glycemia. PBM also showed positive effects on lipid profiles with a reduction in free fatty acid, triglycerides and cholesterol levels. In the clinical study (79), PBM applied on eight muscles in patients with T2D resulted in a decrease in glycemia (fasting and 1h post-prandial glucose) observed 30 min to 12 h after PBM treatment. This decrease was evidenced by a reduction in the GTT AUC by 37% and a reduction of 1h postprandial glucose by 16%. Importantly, no additive effect of hypoglycemic treatment was observed and no hypoglycemia were reported.

**Table 4:** Effect of PBM on glucose metabolism in T2D.

Author/Year			Model		Wave (nm)		Light		PBM parameters		Conclusions
Min 2022 (70)			Diabetic Goto Kakizaki rat		630		Continuous LED		3.7mW/cm², 600sec, 2.22J/cm² Frequency: 1 session Site: intra-duodenal		No effect
					630 + 850		Continuous LED		32.72mW/cm², 100sec, 3.6J/cm² Frequency: 1 session Site: intra-duodenal		↓ glucose intolerance at 4 weeks (↓GTT AUC about 14.5%) ↓ insulin resistance only at 1 week (↓HOMA-IR about 22%) ↑ expression of insulin in beta cells
Bonifacio 2021 (71)			C57BL/6 mice High fat diet		808		Continuous laser		3.57W/cm², 8sec, 30J/cm² Frequency: 3 times a week, for 4 weeks Site: 1, direct contact with skin in pancreas region		No effect on glucose tolerance (GTT) No effect on insulin resistance (ITT) No effect on fasting glycemia No effect on body weight No effect on pancreas morphology No effect on adiposity tissue No effect on pancreas morphology
Gong 2021 (72)			C57BL/6 mice High fat diet and mice C57BLK5 diabetic		635		Continuous laser		72.1mW/cm², 10min, 43.3J/cm² Frequency: once a day, for 10 weeks Sites: 2, direct contact with skin		↓ glucose intolerance (↓GTT AUC about 12%) ↑ insulin sensitivity (↓ITT AUC about 10%) ↓ fed glycemia (500 vs. 280mg/dL) ↓ fasted glycemia (490 vs. 200mg/dL) ↑ glycogen in muscle ↓ ectopic fat in muscle ↓ triglycerides and free fatty acid
Gong 2020 (73)			C57BL/6 mice High fat diet		635		Continuous laser		72.1mW/cm², 10min, 43.3J/cm² Frequency: once a day, for 10 weeks Site: abdomen, direct contact with skin		↓ triglycerides, plasmatic FFA ↑ relative oxygen species
Guo 2020 (74)			C57BL/6 mice High fat diet		635		Continuous laser		72.1mW/cm², 10min, 43.3J/cm² Frequency: once a day, for 8 weeks Site: abdomen, direct contact with skin		↓ glucose intolerance (GTT) ↑ insulin sensitivity (ITT) Protect against obesity (weight similar to control) Protect against hyperglycemia (glycemia similar to control) ↓ weight, glycemia, triglycerides, cholesterol, insulinemia ↓ hepatic steatosis
Silva 2020 (76)			Swiss albinos mice High fat diet		630		Continuous LED		779.53mW/cm², 40sec, 31.18J/cm² Frequency: 5 days per week, for 4 weeks Sites: 5, direct contact with skin		↓ glucose intolerance (↓ GTT AUC about 28%) ↓ fasting hyperinsulinemia (↓insulin concentration by 3)
Silva 2018 (77)			Swiss albinos mice High fat diet		780		Continuous laser		259mW/cm², 40sec, 10J/cm² Frequency: 5 days per week, for 4 weeks Sites: 5, direct contact with skin		↓ glucose intolerance (↓ GTT AUC about 16%) No effect on insulin resistance (HOMA-IR) ↓ fatty mass epididymal ↓ total cholesterol ↑ insulin signaling pathway
Yoshimura 2016 (78)			C57BL/6 mice High fat diet		843		Continuous LED		19mW/cm², 300sec, 5.7J/cm² Frequency: day 1, 3, 7, 10, 14 and 21 Site: abdomen, direct contact with skin		↓ glycemia (98 vs. 118 mg/dL for non-treated group) No effect on weight ↓ abdominal fatty infiltration
Scontri 2023 (79)			Human, n = 10 T2D RCT^†^		830		Continuous LED		114.28mW/cm², 50 or 120sec, 5.71 or 13.71J/cm² Frequence: One session and 7 days of washout Sites: 8, muscles, in contact with skin		Effect only with 5.71J/cm² ↓ post prandial glycaemia (30 min to 12h after PBM) Better effect on glycemic control than hypoglycemic treatments ↓ GTT AUG around 37% Faster glucose decay post prandial (16%, -60 vs. -70mg/dL/h) No additive effect with hypoglycemic treatments

^†^: PBM in addition to the best standard of care, at the investigator discretion choice, AUC, Area under the curve; FFA, Free fatty acid; GTT, glucose tolerance test; HOMA-IR, Homeostasis model assessment insulin resistance; ITT, insulin tolerance test; LED, Light emitting diode; PBM, photobiomodulation; RCT, randomized controlled trial; T2D, Type 2 diabetes.↑, increse; ↓, decrease.

### 3.5 Impact of PBM on exercises and muscles in T2D

A total of 7 studies were conducted, including 3 studies on preclinical models (41, 80, 81) and 4 studies in humans (42, 43, 82, 83) (**Table 5**). In preclinical models, PBM has been demonstrated to have a positive impact on biochemical parameters, such as a decrease in oxidative stress and an increase in antioxidant activity. However, in clinical studies focusing on muscular performance and functionality during or after exercise, PBM has failed to show any improvement. No significant effects on the muscular performance were observed.

**Table 5:** Effect of PBM on exercise and muscle in T2D.

Author/Year	Model	Wave (nm)	Light	PBM parameters	Conclusions
da Silva Tonetto 2023 (80)	Wistar rat Streptozotocin and diet induced	660	Continuous laser	571mW/cm², 36.75sec, 21J/cm² Frequency: 5 days per week, for 6 weeks Sites: 2, medium and laterally of gastrocnemius	↓ oxidative activity ↑ antioxidative activity (↑super oxide dismutase)
de Oliveira 2019 (41)	Wistar rat Streptozotocin induced	660	Continuous laser	250mW/cm², 16sec, 2J/cm² Frequency: 3 times a week, for 3 weeks Site: 1, dorsal	No effect on glucose concentration No effect on muscle parameters if PBM was not associated with exercise
Frigero 2018 (81)	Wistar rat Streptozotocin induced	808	Continuous laser	107.1mW/cm², 44sec, 4.71J/cm² Frequency: 1/session of exercise Sites: 3, gastrocnemius	↓ oxidative stress (↓ lactate, ↓ catalase, ↑ supe oxide dismutase) ↑ VO_2_ max and speed of run
Linares 2022 (42)	Humain, n = 13 DT2 RCT^T^	850	Continuous LED	375mW/cm², 140 to 1120 sec, 52.5-420J/cm² Frequency: 1/session of exercise Sites: 7, oblique and rectus abdomen, quadriceps femoris, triceps, hamstrings bilateral	↓ glycemia and lactate 15min after PBM Improvement of cardiac parameters
Gobbi 2021 (82)	Humain, n = 17 DT2 RCT^ø^	620	Continuous LED	52.86mW/cm², 96sec, 5.074J/cm² Frequency: once a day, for 3 days Sites: 4, ankle flexor and extensor bilaterally	No impact on muscular performance No impact on muscular functionality
		940	Continuous LED	33.7mW/cm², 106sec, 3.572J/cm² Frequency: once a day, for 3 days Sites: 4, ankle flexor and extensor bilaterally	No impact on muscular performance No impact on muscular functionality
		620 + 940	Continuous LED	Same parameters of 2 others	No impact on muscular performance No impact on muscular functionality
Milan-Mattos 2020 (43)	Humain, n = 7 T2D RCT^T^	850	Continuous LED	375mW/cm², 40sec, 15J/cm² ou 80sec, 30J/cm² Frequency: 1/session of exercise Sites: 2, quadriceps and triceps bilaterally	No impact on baroreflex during or after exercise No impact of PBM on cardiovascular autonomic control
Francisco 2019 (83)	Humain, n = 16 T2D RCT^T^	850	Continuous LED	375mW/cm², 40sec, 15J/cm² Frequency: 1/session of exercise Sites: 2, quadriceps and triceps bilaterally	No impact of PBM on lactate concentration No impact on cardiopulmonary and hemodynamic adjustments

T, no other treatment for pain; ø, no information provided about other treatment during the study; LED, light emitting diode; PBM, photobiomodulation; RCT, Randomized controlled trial; T2D, Type 2 diabetes.↑, increse; ↓, decrease.

### 3.6 Impact of PBM on healing process

#### 3.6.1 Wound healing

A total of 44 studies were conducted to evaluate impact of PBM on wound healing, including 31 studies on preclinical models (84–114) and 13 studies on humans (45, 47, 48, 115–124) (**Table 6**). In preclinical models, PBM had a predominantly positive effect on wound healing in 94% of studies. PBM improved various aspects of wound healing, including collagen density, fibroblast proliferation, angiogenesis, granulation tissue formation, and epithelialization. These effects were often accompanied by a decrease inflammatory marker. Only one study, which poorly described PBM parameters, did not report a positive effect of PBM (104). Most studies utilized wavelengths in the red to near-infrared spectrum, whereas studies investigating green wavelengths did not report positive effects of PBM (84, 94). In clinical studies, the majority (92%) reported a positive effect of PBM on chronic ulcers healing. Among the 12 RCTs, there was an increase in wound closure ranging from 15% to 47.3% in the control group compared to 37% to 90.8% in the PBM group. Consequently, the PBM groups had smaller wound areas compared to the control groups, with measurements of 2.39 cm² vs. 8.43 cm² (120), indicating a decrease in wound area of approximately 3.2 cm² vs. 10.4 cm² (122). The only study that did not report a positive effect of PBM utilized the shortest exposure time (less than one second) (121). Two studies specified that PBM did not have any reported side effects (115, 117) while other studies did not explicitly mention it.

**Table 6:** Effect of PBM on wound healing and ulcer.

Author/Year		Model		Wave (nm)		Light		PBM parameters		Conclusion
Dungel 2023 (84)		C57BL diabetic mice		629		Pulsed LED		40mW/cm², 360sec, 14.4J/cm² Frequency: day 0 and 1 Site: near to the wound		↑ wound closure ↑ angiogenesis
				540		Pulsed LED		40mW/cm², 360sec, 14.4J/cm² Frequency: day 0 and 1 Site: near to the wound		↑ wound closure ↑ angiogenesis
				470		Pulsed LED		40mW/cm², 360sec, 14.4J/cm² Frequency: day 0 and 1 Site: near to the wound		No effect
Ebrahimpour-Malekshah 2023 (85)		Wistar rat Streptozotocin induced		890		Pulsed laser		20mW/cm², 200sec, 1.08J/cm² Frequency: once a day, for 14 days Sites: 9, direct contact		↑ granulation tissue formation ↓ neutrophils, ↑ macrophages ↑ fibroblasts ↑ vascularization (VEGF)
Mehrvar 2021 (86)		Diabetic mice		670		Continuous LED		60mW/cm², 90sec, 4.5J/cm² Frequency: 5 days per week, for 2 weeks Site: 1, next to the wound		↓ wound area ↓ oxidative stress ↑ Red-Ox ratio
Ahmadi 2020 (87)		Wistar rat Streptozotocin induced		890		Pulsed laser		1mW/cm², 200sec, 0.2J/cm² Frequency: once a day, for 14 sessions Sites: 9, next to the wound		↑ wound healing ↓ inflammation (neutrophils) ↑ fibroblasts ↑ vascular length
Bagheri 2020 (88)		Wistar rat Streptozotocin induced		890		Continuous laser		1mW/cm², 300sec, 0.324J/cm² Frequency: once a day, for 7 days Site: 1, next to the wound		↓ inflammation (macrophages, neutrophiles) ↑ fibroblast
Kouhkeil 2019 (89)		Rat Streptozotocin induced		890		Continuous laser		1.08mW/cm², 200sec, 0.2J/cm² Frequency: 6 days per week, for 2 weeks Site: 1, next to the wound		↓ mast cells ↓ CFU ↑ wound strength
Fekrazad 2018 (90)		Wistar rat Streptozotocin induced		660		Continuous laser		30mW*, 33sec, 2J/cm² Frequency: every 2 days, for 10 days Site: near to the wound		No effect
				810		Continuous laser		200mW*, 5sec, 2J/cm² Frequency: every 2 days, for 10 days Site: near to the wound		No effect
				660 + 810		Continuous laser		Same parameters		↓ TGF-β1
Asghari 2017 (91)		Wistar rat Streptozotocin induced		890		Pulsed laser		0.324J/cm² Frequency: 6 days per week, for 2 weeks Site: 12, next to the wound		↑ wound healing ↓ CFU
Leite 2017 (92)		Wistar rat Alloxan induced		660		Continuous laser		1W/cm², 9 or 130sec, 10 or 140J/cm² Frequency: once a day, for 3 days Site: 1, next to the wound		140J/cm² > 10J/cm² ↑ wound healing ↑ mast cells number, VEGF, FGF, neovascularization ↓ leukocytes number
Fahimipour 2016 (93)		Albinos mice Streptozotocin induced		632.8		Continuous laser		250mW/cm², 16sec, 4J/cm² Frequency: once a day, for 14 days Sites: 2, next to the wound		632.8 > 830 to improve healing ↑ density of collagen fibers ↑ number of fibroblasts ↑ neovascularization
				830		Continuous laser		250mW/cm², 16sec, 4J/cm² Frequency: once a day, for 14 days Sites: 2, next to the wound		=
Fekrazad 2015 (94)		Wistar rat Streptozotocin induced		425		Continuous laser		55mW/cm², 36sec, 2J/cm² Frequency: day 0, 1, 2, 4, 6, 8 Site: 1, next to the wound		Red > Blue & green ↑ wound healing
				532		Continuous laser		50mW/cm², 40sec, 2J/cm² Frequency: day 0, 1, 2, 4, 6, 8 Site: 1, next to the wound		=
				630		Continuous laser		50mW/cm², 40sec, 2J/cm² Frequency: day 0, 1, 2, 4, 6, 8 Site: 1, next to the wound		=
Dancáková 2014 (95)		SD rat Streptozotocin induced		810		Continuous laser		30mW/cm², 30sec, 0.9J/cm² Frequency: once a day, for 7 days Site: 1, next to the wound		↑ wound healing ↑ wound tensile & strength ↑ granulation tissue
Aparecida da Silva 2013 (96)		Wistar rat Streptozotocin induced		660		Continuous laser		1.43W/cm², 80sec, 4J/cm² Frequency: one session Site: 1, next to the wound		↑ collagen density ↓ MMP2 and MMP9
Fathabadie 2013 (97)		Wistar rat Streptozotocin induced		890		Pulsed laser		1.08mW/cm², 200sec, 0.2J/cm² Frequency: once a day, for 6 days Sites: 18, next to the wound		↑ mast cells
Firat 2013 (98)		Wistar rat Streptozotocin induced		940		Continuous laser		1.1W/cm², 9sec, 10J/cm² Frequency: every 2 days, for 7 days Site: 1, next to the wound		↓ inflammation ↑ collagen synthesis ↑ fibroblasts
Dadpay 2012 (99)		Wistar rat Streptozotocin induced		890		Pulsed laser		1.08mW/cm², 30 or 300sec, 0.03 or 0.2J/cm² Frequency: 6 days per week, for 2 weeks Sites: 18, next to the wound		↑ Enhancing maximum stress and elastic modulus
Park 2012 (100)		SD rat Streptozotocin induced		980		Continuous laser		232.5mW/cm², 60sec, 13.95J/cm² Frequency: once a day, for 14 days Site: 1, next to the wound		↓ inflammation cells infiltration ↑ number of fibroblasts ↑ wound healing
Hegde 2011 (101)		Swiss Albinos mice Streptozotocin induced		632.8		Continuous laser		4.02mW/cm², 255 to 1277sec, 1 to 5J/cm² Frequency: once Site: 1, next to the wound		The best = 3J/cm² ↑ wound healing ↑ collagen synthesis
Peplow 2011 (102)		Diabetic mice		660		Continuous laser		233-313mW/cm², 20sec, 2J* 116-156mW/cm², 40 sec, 2J* 58-78mW/cm², 80sec, 2J* Frequency: once a day, for 7 days Site: 1, next to the wound		Same effects between puissance ↑ wound healing ↑ epithelialization, granulation
Akyol 2010 (104)		Wistar rat Streptozotocin induced		808		Continuous laser		100mW/cm², 20sec, 2J/cm² Frequency: every 2 day, for 8 days Site: 1, next to the wound		↑ wound healing No effect on inflammation No effect on epithelialization
Carvalho 2010 (103)		Wistar rat Alloxan induced		660		Continuous laser		166mW/cm², 24sec, 4J/cm² Frequency: unknown Site: 1, next to the wound		↑ fiber of collagen ↓ macrophages
Chung 2010 (105)		Diabetic mice		660		Continuous laser		10sec -> 1J*, 20sec -> 1.6J*, 40sec -> 3.2J* Frequency: once a day, for 7 days Site: 1, next to the wound		Best one = 1.6J/day ↑ wound healing
Santos 2010 (106)		Wistar rat Streptozotocin induced		660		Continuous laser		30mW* -> 2,5J/cm² Frequency: once a day, for 8 days Sites: 16, next to the wound		790 better than 660 ↑ angiogenesis
				790		Continuous laser		40mW* -> 2,5J/cm² Frequency: once a day, for 8 days Site: 16, next to the wound		
Al-Watban 2009 (107)		SD rat Streptozotocin induced		532		Continuous laser		20.4mW/cm², 290 to 1470sec, 5 to 30J/cm² Frequency: 3 times per week		Best = Laser 633 ↑ wound healing
				633		Continuous laser		15.56mW/cm², 322 to 1932sec, 5 to 30J/cm² Frequency: 3 times per week		
				810		Continuous laser		22.2mW/cm², 225 to 1350sec, 5 to 30J/cm² Frequency: 3 times per week		
				980		Continuous laser		22.2mW/cm², 225 to 1350sec, 5 to 30J/cm² Frequency: 3 times per week		
				1060		Continuous laser		66.37mW/cm², 75 to 450sec, 5 to 30J/cm² Frequency: 3 times per week		
				510-872		Continuous LED		13.6mW/cm², 367 to 2206sec, 5 to 30J/cm² Frequency: 3 times per week This was a polychromatous LED		
Güngörmüş 2009 (108)		Wistar rat Streptozotocin induced		808		Continuous laser		10J/cm² Frequency: every 2 days for 8 days Site: unknown		↑ wound healing
Maiya 2009 (109)		Wistar rat Alloxan induced		632.8		Continuous laser		10mW/cm², 3 to 27min, 3 to 9J/cm² Frequency: 5 days per week until wound healing Site: 1, next to the wound		3 to 7J/cm²: ↑ epithelialization, tissue granulation ↑ wound healing 8-9J/cm²: ↓ reparative process
Carvalho 2006 (110)		Wistar rat Alloxan induced		632.8		Continuous laser		200mW/cm², 60sec, 4J/cm² Frequency: once a day, for 14 days Site: 1, next to the wound		↑ fiber of collagen
Rabelo 2006 (111)		Wistar rat Streptozotocin induced		632.8		Continuous laser		588mW/cm², 17sec, 10J/cm² Frequency: once a day, for 15 days Site: 1, next to the wound		↓ wound area ↓ local inflammation ↓ inflammatory cells
Maiya 2005 (112)		Wistar rat Alloxan induced		632.8		Continuous laser		4.8J/cm² Frequency: 5 days per week until wound healing Site: 1, next to the wound		↑ collagen ↑ fibroblastic and capillary proliferation ↑ granulation tissue formation, vascularization, epithelialization
Byrnes 2004 (113)		Purina sand rat chow 5L09 DT2 model		632.8		Continuous laser		16mW/cm², 250sec, 4J/cm² Frequency: once a day, for 3 days Site: 1, next to the wound		↑ wound closure ↑ collagen, bFGF ↑ neovascularization
Reddy 2001 (114)		SD rat Streptozotocin induced		632.8		Continuous laser		1J/cm² Frequency: once a day, for 5 days Site: 1, next to the wound		↑ collagen ↑ maximum strain ↑ toughness
Haze 2022 (115)		Human, n = 20 Diabetes RCT^†^		808		Continuous laser		138mW/cm², 8min, 1.1J/cm² Frequency: once a day, for 12 weeks Site: next to the wounds		↓ wound area (12.5 vs. 1.5cm²) ↑ wound closure (49.4 vs. 97.3%) No side effects link to PBM
Vitoriano 2019 (116)		Human, n = 12 Diabetes RCT^†^ (for 2 sources of light)		850		Continuous LED		240mW/cm², 22sec, 14.64J/cm² Frequency: 2 times a week, for 5 weeks Sites: 6, next to the wound		Laser seems better than LED ↓ wound area (1.45 to 0.64 vs 1.76 to 0.36cm²)
				830		Laser		250mW/cm², 28sec, 15.48J/cm² Frequency: 2 times a week, for 5 weeks Sites: 3, next to the wound		
de Alencar Fonseca Santos 2018 (45)		Human, n = 18 Diabetes RCT^†^		660		Continuous laser		490mW/cm², 13sec, 6J/cm² Frequency: every 2 days, for 4 weeks Site: 1, next to the wound		↑ wound healing index ↑ pressure ulcer scale for healing No effect on pain (VAS)
Frangez 2018 (117)		Human, n = 60 Diabetes RCT^†^		625 (24%) 660 (71%) 850 (5%)		Pulsed LED		5min, 2.4J/cm² Frequency: 3 times a week, for 8 weeks Site: 1, next to the wound		↑ Falanga score (score of healing) No effect on size
Ruh 2018 (118)		Human, n = 8 Diabetes Pre-Post ITV^†^		660		Continuous laser		167mW/cm², 12sec, 2J/cm² Frequency: once a day for 12 days Site: 1, next to the wound		↓ wound size (data not shown) ↓ TNFα, ↑TGFβ, ↑VEGF No effect on IL6
Mathur 2017 (119)		Human, n = 30 T2D RCT^†^		660		Continuous laser		50mW/cm², 60sec, 3J/cm² Frequency: once a day, for 15 days Sites: 5-8, above the wound		↑ wound closure (15% vs. 37%) No side effects
Carvalho 2016 (120)		Human, n = 32 DT2 RCT^†^		658		Continuous laser		50mW/cm², 80sec, 4J/cm² Frequency: 3 times a week, for 4 weeks Site: 1, next to the wound		↓ wound area (8.43 vs. 2.39cm²) ↓ pain (VAS: 4.8 vs 1.9) ↑ neovascularization
Sandoval Ortíz 2014 (121)		Human, n = 9 Diabetes RCT^†^		685		Continuous laser		~11mW/cm², 0.14-0.18sec, 1.5-2J/cm² Frequency: Unknown Sites: multiple along the edges of the ulcer and in the wound bed, next to the wound		No effect on wound healing No effect on protective sensation No effect on QoL (EQ-5D)
Kajagar 2012 (122)		Human, n = 68 T2D RCT^†^		660 + 850		Pulsed LED		60mW*, 2-4J/cm² Frequency: once a day, for 15 days Site: 1, above the wound		↓ ulcer area (decrease of 32 vs. 104cm²)
Kaviani 2011 (123)		Human, n =23 T2D RCT^†^		685		Continuous laser		50mW/cm², 200sec, 10J/cm² Frequency: 6 days per week, at least 2 weeks Site: 1, next to the wound		↑ wound closure (47.3% vs 73.7% after 4 weeks) ↑ wound healing (non-ischemic wound)
Minatel 2009 (124)		Human, n = 14 Diabetes RCT^†^		890 + 660		Continuous LED		100mW/cm², 30sec, 3J/cm² Frequency: 2 times a week, for 3 months Site: 1, next to the wound		↑ granulation ↑ wound closure (43.3% vs. 90.8%)
Schindl 1999 (47)		Human, n = 8 Diabetes Descriptive^†^		632.8		Continuous laser		30mW*, 30J/cm² Frequency: 3 times a week until wound healing Site: unknown		100% of closure of chronic ulcers after 32 to 130 sessions
Schindl 1998 (48)		Human, n = 30 Diabetes RCT^†^		632.8		Continuous laser		10mW/cm², 50min, 30J/cm² Frequency: 1 time Site: 1, skin surface		↑ skin temperature ↑ microcirculation in patient with microangiopathy

^†^: PBM in addition to the standard wound care (rising, cleaning, drying), CFU, colony forming unit; FGF, fibroblast growth factor; LED, Light emitting diode; MMP, Matrix metalloproteinases; PBM, Photobiomodulation; Pre-Post ITV, Pre-post interventional trial; QoL, Quality of life; Rat SD, Rat Sprague Dawley; RCT, Randomized controlled trial; T2D, Type 2 diabetes; TGF, Transforming Growth factor; TNF, Tumor necrosis factor; VAS, Visual analogic scale; VEGF, Vascular endothelial growth factor. *Surface of PBM not reported.↑, increse; ↓, decrease; =, equal/same.

#### 3.6.2 Healing of bone defect

A total of 16 studies were conducted to evaluate impact of PBM on healing of bone defect, with 15 studies conducted on preclinical models (125–139) and one study in humans (140) (**Table 7**). Among these studies, only one preclinical study did not report a positive impact of PBM on bone repair. This study used the highest power among all studies, with a dosage of 369.4J/cm². In addition to the effect of PBM on bone repair, several studies reported an increase in bone vascularization and a decrease in inflammation.

**Table 7:** Effect of PBM on healing of bone defect.

Author/Year		Model		Wave (nm)		Light		PBM parameters				Conclusions		
Dalirsani 2021 (125)		Wistar rat Streptozotocin induced		660		Continuous laser		76.4mW/cm², 24sec, 7.2J/cm² Frequency: once a day, for 14 days Site: 1, direct contact				↑ bone formation ↓ inflammation ↑ vascularization		
				802		Continuous laser		127.32mW/cm², 14sec, 7J/cm² Frequency: once a day, for 14 days Site: 1, direct contact				↓ inflammation ↑ vascularization		
Lee 2021 (126)		Wistar rat Streptozotocin induced		660		Continuous laser		2.42mW/cm², 1652sec, 4J/cm² Frequency: once a day, for 12 weeks Site: 1, near to the bone defect place				↑ bone formation ↑ bone fracture healing No effect on osteogenic factor		
Diker 2019 (127)		SD rat Streptozotocin induced		808		Continuous laser		3.5W/cm², 22sec, 78.5J/cm² Frequency: once a day, for 3 days Site: 1, direct contact				↑ bone formation ↑ osteoblasts		
Gomes 2018 (128)		Wistar rat Streptozotocin induced		780		Continuous laser		16W/cm², 10, 20 or 40sec, 160, 320 or 640J/cm² Frequency: every 2 days, for 21 days Site: 1, direct contact				Only or 640J/cm²: Better maintenance of periodontal tissue subjected to a force		
Mostafavinia 2018 (129)		Wistar rat Streptozotocin induced		890		Pulsed laser		1.5W/cm², 1300sec, 1.5J/cm² Frequency: 3 times a week, for 4 weeks Sites: 3, direct contact				↑ bone formation ↑ bone cortical volume ↑ bone trabecular volume ↑ osteoblasts and osteocytes		
Mostafavinia 2017 (130)		Wistar rat Streptozotocin induced		890		Pulsed laser		8.32W/cm², 1300sec, 1.5J/cm² Frequency: 3 times a week, for 4 weeks Sites: 3, direct contact				↑ bone density		
Yildirimturk 2017 (131)		SD rat Streptozotocin induced		820		Continuous laser		0.5W/cm², 32sec, 16J/cm² Frequency: 3 times a week, for 4 weeks Site: 1, tibiae, direct contact				↑ bone formation ↑ vascularization No effect of osteoblast quantity		
Patrocínio Silva 2016 (132)		Wistar rat Streptozotocin induced		808		Continuous laser		3.57W/cm², 33sec, 120J/cm² Frequency: 3 times a week, for 8 weeks Site: 1, direct contact				↑ bone density ↑ bone mineral content stiffness ↑ cortical tibia area		
Magri 2015 (133)		Wistar rat Streptozotocin induced		808		Continuous laser		3.57mW/cm², 8 or 16 or 33sec, 30, 60 or 120J/cm² Frequency: 3 times a week, for 4 weeks Sites: 2, direct contact				↑ bone formation No histological effect		
Nascimento 2015 (134)		Wistar rat Alloxan induced		780		Continuous laser		1.75W/cm², 10sec, 17.5J/cm² Frequency: every 2 days, for 7 days Site: 1, direct contact				↑ bone formation ↓ inflammation ↑ alkaline phosphatase		
Patrocínio Silva 2014 (135)		Wistar rat Streptozotocin induced		808		Continuous laser		3.57W/cm², 33sec, 120J/cm² Frequency: 3 times a week, for 6 weeks Sites: 4, direct contact				↑ bone density ↑ cortical area ↑ values of fracture force ↑ osteogenic potential		
Akyol 2010 (136)		Wistar rat Streptozotocin induced		808		Continuous laser		100mW/cm², 20sec, 2J/cm² Frequency: every 2 days, for 7 days Site: 1, right distal epiphysis				↑ bone repair ↑ substantia spongia formation No effect on union bone marrow		
Abdi 2009 (137)		Wistar rat Alloxan induced		780		Continuous laser		318mW/cm², 1166sec, 369,4J/cm² Frequency: 3 times a week, for 6 weeks Sites: 2, direct contact				No effect on bone repair		
Bayat 2009 (138)		Wistar rat Streptozotocin induced		632.8		Continuous laser		3.17mW/cm², 90 or 1200sec, 88.6 or 382.2J/cm² Frequency: once a day, for 14 days Sites: 4, direct contact				↑ bone density ↑ bone lamella meshwork ↑ maximum force and load at the break ↓ bend stiffness		
Javadieh 2009 (139)		Wistar rat Streptozotocin induced		890		Pulsed laser		265 or 530sec, 5 or 10J/cm² Frequency: 3 times a week, for 6 weeks Sites: 2, direct contact				↑ bone repair ↑ bending stiffness ↑ maximum force		
Attia 2023 (140)		Human, n = 40 T2D RCT^ø^		808		Continuous laser		125mW/cm², 1.23min, 0,15J/cm² Frequency: 2 times, pre and post implantation Sites: 6, direct contact				↑ bone repair and density ↑ bone structure		

^ø^: no information provided about other treatment during the study. Rat SD, Rat Sprague Dawley; RCT, Randomized controlled trial; T2D, Type 2 diabetes.↑, increse; ↓, decrease.

#### 3.6.3 Chronic periodontitis

A total of 16 studies were conducted to evaluate the impact of PBM on chronic periodontitis in humans (34–40, 141–149) (**Table 8**).

**Table 8:** Effect of PBM on chronic diabetes periodontitis.

Author/Year	Model	Wave (nm)	Light	PBM parameters	Conclusions
Kamatham 2022 (34)	Human, n = 60 T2D RCT^T^	650	Continuous laser	0.4W* Frequency: 1 session Site: 1/tooth, in contact with gingival tissue	↓ inflammation ↓ calprotectin No effect on probing depth, clinical attachment level
Pulivarthi 2022 (35)	Human, n = 30 T2D RCT^†^	650	Continuous laser	0.8W/cm², 15sec, 12J/cm² Frequency: once a day, for 8 weeks Sites: 3, in contact with gingival tissue	No effect on TNFα No effect on bleeding index, probing depth, clinical attachment level
Mrasori 2021 (141)	Human, n = 80 T2D RCT^†^	660	Continuous laser	10mW*, 8min Frequency: 5 days per week, for 3 months Sites: 5, in contact with gingival tissue	↓ IL6
Soi 2021 (142)	Human, n = 44 T2D RCT^†^	940	Pulsed laser	0.8W*, 15sec, 24J* Frequency: unknown Sites: 2/tooth, into the periodontal pocket	No effect of adjunction of PBM to SRP (scaling and root planning)
Koçak 2020 (36)	Human, n = 60 T2D RCT^†^	940	Pulsed laser	1.061W/cm², 20sec Frequency: 1 session Sites: 2/tooth, intra periodontal pocket	No effect on bacteria level (*P.gingivalis*, *T.forsythia*, *T.denticola*)
Özberk 2020 (143)	Human, n = 22 T2D RCT^†^	980	Continuous laser	33mW/cm², 15sec, 0.5J/cm² Frequency: day 0, 1, 3 and 7 Sites: 2/tooth, in contact with maxilla and mandibula	↓ probing depth (2.9 vs. 2.6 mm) ↓ clinical attachment level (3.0 vs. 2.8 mm) No effect on plaque index and gingival index
Castro dos Santos 2019 (144)	Human, n = 24 T2D RCT^†^ (on pocket)	660	Continuous laser	1.1W/cm², 20sec, 22J/cm² Frequency: 1 session Sites: 2, buccal and lingual	No effect on probing depth, clinical attachment level
Chandra 2019 (145)	Human, n = 40 T2D RCT^†^	808	Continuous laser	1.5-1.8W/cm², time in second Frequency: 1 session Site: 1, intra periodontal pocket	↓ plaque index (1.56 vs. 1.26) ↓ gingival index (1.56 vs. 1.04) ↓ probing depth (2.63 vs. 1.80) ↓ clinical attachment level (7.50 vs. 6.65) ↓ bacteria level (35% more reduction with PBM)
Dengizek Eltas 2019 (146)	Human, n = 40 T2D RCT^†^	810	Continuous laser	1W*, 15-20sec Frequency: once a day Sites: 3/tooth	↓ gingival index (0.91 vs. 0.58) ↓ bleeding on probing (31.7 vs. 24.7%) ↓ probing depth (2.99 vs. 2.77mm) No effect on plaque index, clinical attachment level and inflammation (CRP)
Li 2018 (147)	Human, n = 80 T2D RCT^†^	Unknown	Unknown	Unknown	↓level of TNF, IL-1, LPS Increase leptin
Demirturk-Gocgun 2017 (148)	Human, n = 22 T2D RCT^†^ (on pocket)	808	Continuous laser	0.89W/cm², 5sec, 4.46J/cm² Frequency: Day 1, 2 and 7 Sites: 4, in contact with gingival tissue	No effect on bleeding of probing, probing depth, clinical attachment level, plaque index
Koçak 2016 (37)	Human, n = 60 T2D RCT^†^	940	Pulsed laser	1.061W/cm², 20sec Frequency: 1 session Sites: 2/tooth, intra periodontal pocket	↓ VCAM No effect on IL1/6/8/ICAM
Javed 2015 (149)	Human, n = 22 T2D RCT^†^ (on pocket)	1064	Pulsed laser	1430W/cm², 60 to 120sec (depending of the accessibility of the pocket), 240-480J* Frequency: unknown Site: 1/tooth, into the periodontal pocket	↓ plaque index (6.4 vs. 1.5) at 1 month, not 3 months ↓ bleeding probing (5.5 vs. 2.1) at 1 month, not 3 months
Obradović 2013 (38)	Human, n = 300 T1D, T2D RCT^†^	670	Continuous laser	2mW/cm², 16min, 2J/cm² Frequency: once a day for 5 days Site: 1, in contact with gingival tissue	↓ alteration of periodontium (histologic description)
Obradović 2012 (39)	Human, n = 200 T1D, T2D RCT^†^ (on pocket)	670	Continuous laser	5mW*, 14min Frequency: once a day, for 5 days Site: 1, in contact with the jaws	↓ gingival index (0.31 vs. 0.16) ↓ inflammation ↑ cytomorphometric parameters
Obradović 2011 (40)	Human, n = 150 T1D, T2D Pre-Post ITV^†^	Unknown	Unknown	5mW* Frequency: for 5 days Site: only right site of the jaw	↓ gingival index (data not available) ↓ nuclei areal

T: no other treatment for pain, ^†^: PBM in addition to non-surgical periodontal treatment (i.e. scaling and root planning, ultrasonic periodontal debridement), CRP: C reactive protein, ICAM: Intercellular adhesion molecule, IL: Interleukin, LPS: lipopolysaccharide, PBM: photobiomodulation, Pre-post ITV: Pre-post interventional trial, RCT: Randomized controlled trial, SRP: scaling and root planning, T1D: Type 1 diabetes, T2D: Type 2 diabetes, TNF: Tumor necrosis factor, VCAM: Vascular cell adhesion molecule, *Surface of PBM not reported.↑, increse; ↓, decrease.

▪ Effect on healing process

The effects of PBM were evaluated using the following measures:

1) gingival index represents inflammation of the gingival tissue (150),
2) the plaque index, which represents the presence of supragingival plaque on all four tooth surfaces (151).

Among the clinical studies, 11 were RCTs, 3 were RCTs specifically focused on pockets treated with PBM, and two were Pre-Post ITV. The results were heterogeneous, with a decrease in the plaque index observed in 50% of the studies, a decrease in the gingival index in 80% of the studies, a reduction in bleeding in 66% of the studies, a decrease in probing depth in 50% of the studies, and improvements in clinical attachment levels in 29% of the studies.

▪ Effect on bacterial population

Two studies investigated the effect of PBM on reducing the bacterial population at periodontitis sites, but the results were contradictory.

### 3.7 Others utilization described in diabetes mellitus

#### 3.7.1 Erectile function

In a preclinical study, Yang et al. (152) reported a positive impact of PBM on erectile function two weeks after PBM exposure. This suggests a potential therapeutic effect of PBM on improving erectile function (**Table 9**).

**Table 9:** Effect of PBM on other complications.

Author/Year	Model	Wave (nm)	Light	PBM parameters	Conclusions
Yang 2023 (152)	SD rat Streptozotocin induced	808	Laser	4J/cm² Frequency: for 2 weeks Site: Unknown	↑ erectile function ↑ mitochondrial function and morphology ↓ oxidative stress
Asghari 2016 (153)	Wistar rat Streptozotocin induced	685	Continuous laser	53.6mW/cm², 60sec, 3.2J/cm² Frequency: H0, H1, H2 Sites: 6, direct contact with skin	↓ ischemia-reperfusion injury ↓ plasma creatinine ↓ tubular dilatation, glomerular atrophy ↑ glutathione, superoxide dismutase and catalase
Aghamohamdi 2020 (154)	Human, n = 30 Diabetes Pre-Post ITV^†^	830	Pulsed laser	334mW*, 60sec, 16J/cm² Frequency: 3 times a week, for 4 weeks Sites: 9, pathway of facial nerve, direct skin contact	Recovery in electromyogram in diabetic patient with Bell’s palsy
		980	Pulsed LED	9min, 5J/cm² Frequency: 3 times a week, for 4 weeks Sites: 9, pathway of facial nerve, direct skin contact	

^†^: PBM in addition to the best standard of care, LED, Light emitting diode; Pre-Post ITV, Pre-post interventional trial. *Surface of PBM not reported.↑, increse; ↓, decrease.

#### 3.7.2 Ischemia reperfusion injury

Asghari et al. (153) conducted a preclinical study and demonstrated a protective effect of PBM against ischemia/reperfusion injury in the diabetic kidney. They observed a decrease in tubular epithelial necrosis, polymorphonuclear cells in the outer medulla, cellular oedema, tubular dilatation, hyaline casts, and medullary congestion. These findings indicate the potential of PBM in mitigating kidney injury associated with ischemia/reperfusion (**Table 9**).

#### 3.7.3 Facial nerve palsy

Aghemohamdi et al. (154) demonstrated the positive impact of PBM in patients with T2D who experienced facial nerve palsy. After 12 sessions of PBM, 60% of the patients showed recovery on electromyogram without any reported side effects. However, the investigation of QoL outcomes were not investigated in this study (**Table 9**).

## 4 Discussion

This review shows a clear interest in the use of PBM in diabetes, both at preclinical (70 studies) and clinical level [56 studies out of 88 clinical trials identified by clinicaltrials.gov (27)]. However, the therapeutic effect of PBM is variable, with inconsistent illumination parameters that are not standardized across studies.

Regarding clinical trials, PBM has generated interest across various fields, with 2,573 clinical trials identified on clinicaltrials.gov (27). Although 42 clinical trials related to PBM in diabetes were found on clinicaltrials.gov (27), an additional 46 studies were identified through the Medline bibliographic search. Since PBM is not considered as a drug, the reporting of trials in the global clinical trials database is not consistent. Moreover, in some cases, trials may be reported directly to national registries, as seen in many studies conducted in Brazil. Another important point to note is that among the 67 completed trials, only 56 were published, indicating a significant publication bias (16%), which is likely underestimated. Moreover, quality of clinical trials must be upgraded, as there is heterogeneity in the number of patients included, in the presence of a control group and in the parameters used. Furthermore, in terms of clinical publications, few teams have conducted more than one study, amplifying the heterogeneity of the PBM parameters used.

Currently, there is no consensus on the optimal PBM parameters to achieve biological or clinical effects. In terms of light sources, some studies have reported superior effects with coherent laser light (155, 156). However, a recent review found no difference in efficacy between LEDs and laser sources, with LEDs being more cost-effective. In the context of diabetes and wound healing, two studies compared lasers and LEDs. Al-Watban et al. (107) reported a better efficacy of a 633 nm laser compared to LEDs with polychromatic light for ulcer healing, may be due to dilution of the effect as the irradiance was comparable for both. Vitoriano et al. (116) reported a greater reduction in ulcer size with an 830 nm laser compared to 850 nm LEDs (with comparable irradiance). Despite these two studies favoring lasers, numerous studies in the field of diabetes have reported positive effects of LED-based PBM. However, the choice between laser and LED was a technologic choice and could be led by the accuracy of wavelength search, the availability of the device and energetic consumption. It is important to note that the principle of PBM is based on the Arndt-Schultz law (20), which describes a biphasic response. A dose that is too low will produce no effect while a dose that is too high can be toxic and induce mitochondrial permeabilization and apoptosis through activation of caspases (157). This biphasic response to PBM was reflected in two studies on ulcer healing in diabetes. Hedge et al. (101) tested a 632.8 nm laser with different irradiances ranging from 1 to 5 J/cm². While irradiances of 1 and 5 J/cm² resulted in poorer and slower wound healing, an irradiance of 3 J/cm² appeared to be optimal for improving and accelerating wound healing. Maiya et al. (109) tested a 632.8 nm laser with different irradiances ranging from 3 to 9 J/cm². Irradiances between 3 and 7 J/cm² had a positive effect on healing, including increased epithelialization, tissue granulation, and accelerated wound healing; whereas irradiances of 8 and 9 J/cm² hindered the healing process. To date, there is no consensus on the power or optimal irradiance to be applied, and the wavelength applied is another parameter of interest that lacks consensus and may depend on the target tissue. Red and near-infrared light correspond to the absorption wavelengths of cytochrome c oxidase in the mitochondria (23). Green light, on the other hand, is rarely used and not very effective in inducing biological changes as it was not in the specter of absorption of cytochrome c oxidase. Two studies compared blue, green, and red wavelengths in diabetes. Dungel et al. (84) reported that blue light (470 nm) had no effect on wound healing, whereas green (540 nm) and red (629 nm) light accelerated wound healing. Fekrazad et al. (94) reported no effect of blue (425 nm) and green (532 nm) light, whereas red light (630 nm) promoted wound healing. Another study (93) reported that a 632.8 nm laser is more effective than an 830 nm laser for wound healing. Given these findings, it is logical that the most commonly used wavelengths (80 studies) fall within the red and near-infrared range (600–810 nm). In any case, numerous articles have shown that the effect of PBM depends on various parameters: wavelength, fluence (J/cm²), total energy received (J), pulsed or continuous emission mode … Moreover, the absorption characteristics of the tissue, as well as the delivery mode, and the frequency of use of the PBM (number of applications, treatment schedule etc.) add complexity. It is now necessary to standardize PBM parameters, and to precise them into papers; in order to be reproducible and identify effective application methods.

The first experiment to investigate the effect of PBM on healing showed promising results (17, 18). Extensive research has been conducted in this field, with several preclinical and clinical reviews reporting positive effects of PBM on wound healing (158), healing of bone defect (159), and periodontitis (160). In a specific population of patients with diabetes, the results regarding these healing processes were encouraging. Numerous preclinical studies have focused on ulcer and wound healing, demonstrating the beneficial effects of PBM. These effects include improved angiogenesis and associated trophic factors, increased fibroblasts, reduced inflammation, increased collagen quantity, and even a reduction in colony-forming units (CFU). Clinically, these results were supported by a significant reduction in wound area (by a factor of 3.5 to 8.2) and increased wound closure (ranging from 22% to 47.9%), which may be associated with reduced pain. Wound healing issues in patients with diabetes significantly impact their QoL (161). However, only one study (121) has examined the impact of PBM on the QoL of patients with diabetes and ulcers and did not demonstrate any beneficial effects of PBM on QoL. Overall, the data on wound healing are encouraging. The ideal parameters may involve repeated exposure over several weeks, at multiple sites as close as possible to the lesion, with a fluence between 1 and 10 J/cm², continuous illumination using LED or laser, and a wavelength ranging from 660 to 830 nm. In wound healing, PBM appeared as a sage approach to enhance healing process in addition to wound standard of care.

Regarding healing of bone defect, 94% of preclinical studies showed positive results (improved vascularization, increased osteoblasts and osteocytes, reduced inflammation, increased bone volumes, and enhanced bone density). Clinically, only one study (140) has been conducted, demonstrating improved bone repair, density, and structure after dental implant insertion with one session of PBM before and after implantation. However, conclusions cannot be drawn from a single clinical study, but the promising results from preclinical and clinical studies should motivate further clinical trials to determine the optimal parameters for PBM.

To date, only clinical studies have investigated the effects of PBM on periodontitis, yielding heterogeneous results due to variations in the applied parameters. The exposure periods ranged from a single session to several days or even weeks. Among studies that examined the gingival index, plaque index, and clinical attachment level, 75% reported improvements in at least one of these parameters. Four studies reported negative results: one had the highest fluence (144), one had the highest exposure frequency [once a day for 8 weeks (35)], one had poorly described illumination parameters and a wavelength beyond the infrared range (142), and one had four PBM exposure sites (148), whereas most studies reported one to two exposure sites. Finally, the ideal parameters could involve a single exposure or exposure over 2 to 3 days, on one to two sites in direct contact with gingival tissue or intra-pocket, with a fluence of 1 to a few J/cm², continuous or pulsed laser illumination, and a wavelength range of 650 to 1064 nm. These data have been supported by previous results on *in vitro* model (162) reporting positive response of fibroblasts to the PBM in diabetic hypoxic wounded models. Even if three teams published several studies, the lack of rigorous methodology and the heterogeneity of PBM parameters, did not allowed to identify leader in this field. Moreover, due to the wide variability in illumination parameters and obtained results, definitive conclusions regarding the therapeutic effects of PBM on periodontitis cannot be formally drawn. Further clinical trials are required to establish clearer conclusions and defined optimal PBM parameters to use PBM as an added therapy for diabetes chronic periodontitis management.

Regarding neuropathy, five preclinical studies reported a positive effect of PBM on pain, leading to a decrease in cytokines and improvement in mitochondrial parameters. Clinically, in 77% of the studies, a 2-5 points reduction on the VAS was observed for pain. Out of the studies that considered the impact of PBM on QoL (24%), two studies reported no effect (58, 59) (similarly, no effect on pain was observed), while two studies reported a 4-point improvement in Norfolk Quality of Life-Diabetic Neuropathy (54) and SF-36 scores on all these parameters (44). No adverse effects of PBM were reported in any of the studies. Two teams have been identified in this field. Unfortunately, studies of Burka et al. (28–30). lack methodological rigor and a description of the PBM parameters used. On the other hand, Arun G et al. (31–33), succeeded in demonstrating in their three studies (with the same PBM parameters used) an improvement of vibration perception threshold, decrease of pain, improvement of microcirculation and biological parameters. These benefits were observable as early as 10 days after daily PBM with 2 lasers (632.8 nm and 660 nm + 880 nm) over 9 min (3.1 J/cm² of fluence) on the plantar and dorsal surfaces of the feet. These parameters should therefore serve as a basis for future clinical trials aimed at defining whether MBP will be used instead of or in addition to current pharmacological treatments.

For diabetic retinopathy, only a few studies have been conducted in this field. Four preclinical studies reported promising results on the effect of PBM, showing histological improvements in the retina. However, clinically, only two studies reported a reduction in central macular thickness, with (69) or without (68) an improvement in vision. Therefore, it is not possible to conclude whether PBM must be used. Clinical trials must be conducted to demonstrate PBM safety in use and its efficacy as a complementary or alternative therapy to current therapeutic options.

PBM is also gaining popularity among its potential benefits for post-physical activity recovery, this fact was still a source of debate (163, 164). In the context of T2D, a limited number of studies (n = 7) have been conducted. The results of 3 preclinical studies reported positive effects of PBM on oxidative stress, antioxidant activity, and muscular parameters. However, clinically (n = 4), no study reported improvements in performance or muscle functionality. Only one study (42) reported a benefit in terms of lactate concentration and cardiac parameters. Based on these findings, it can be concluded that the current parameters used for PBM do not provide benefits for post-exercise muscle recovery in patients with T2D.

Lastly, since diabetes is a metabolic disease characterized by an imbalance in glycemic control, PBM has also been investigated in this field. Preclinical results have shown promising results, with 83% of the studies that examined glucose tolerance possibly due to a direct impact of PBM on islet insulin secretion capability and insulin resistance reporting an improvement in these parameters. The only negative study (71) applied the highest power (3.57 W/cm²) for the shortest period (8 seconds). Preclinical studies have also demonstrated beneficial effects of PBM on lipid profiles, including reduced ectopic fat in muscle, triglycerides, and free fatty acids. Additionally, a small-scale clinical study (79) (n = 10) reported beneficial effects of PBM, including a 37% reduction in post-meal AUC for glucose and approximately 16% faster postprandial glucose decay. No adverse effects were reported with PBM, and there was no increased risk of hypoglycemia when PBM was combined with hypoglycemic treatments. Overall, these findings support the potential of PBM in improving glycemic control in patients with type 2 diabetes. Further clinical trials with larger sample sizes are warranted to determine the optimal parameters for PBM as an additional therapy in the therapeutic arsenal, helping to improve patients’ glycemic control.

Moreover, the effect of PBM on glucose intolerance could be lead to the action of PBM on beta cells and islets.

Liebman et al. (165) reported improvement of insulin secretion of beta cells and glucagon of alpha cells associated with a rise of calcium activity. Irani et al. (166) demonstrated that PBM could improve insulin secretion of rat pancreatic islets with poor insulin secretion. Huang et al. (167) investigated the effects of PBM on pig islets, which are being explored as a potential source of islets for xenotransplantation. However, they did not observe any significant positive or negative effects on glucose-stimulated insulin secretion. Further research is needed to explore the potential of PBM to enhance islet function for transplantation purposes, even if Asghari et al. (153) reported protector effect of PBM on ischemia-reperfusion injury in diabetic kidney of rats.

## 5 Conclusion

Overall, this review highlights the growing interest in PBM as a potential therapeutic approach for various aspects of diabetes. This study emphasizes the potential of PBM as a valuable approach for managing wound healing issues and neuropathic pain in diabetic patients in both preclinical and clinical studies. The potential benefits of PBM in healing of bone defect and glycemic control show promise. In retinopathy, the small number of studies make it impossible to draw any conclusion. In periodontitis, more extensive clinical trials are warranted to establish the optimal parameters and protocols for PBM. Likewise, the current evidence does not support the use of PBM for muscle recovery after physical exercise.

## Author contributions

QP: Writing – original draft, Resources, Methodology, Investigation, Formal analysis, Data curation, Conceptualization. CM: Writing – review & editing, Validation, Supervision, Funding acquisition. SL: Writing – review & editing, Validation, Supervision, Funding acquisition, Conceptualization.

## References

[1] IDF - diabetes atlas (2021). Available at: https://diabetesatlas.org/idfawp/resource-files/2021/07/IDF_Atlas_10th_Edition_2021.

[2] Marcovecchio ML Lucantoni M Chiarelli F. Role of chronic and acute hyperglycemia in the development of diabetes complications. Diabetes Technol Ther. (2011) 13:389−94. doi: 10.1089/dia.2010.0146 21299400

[3] Yamazaki D Hitomi H Nishiyama A. Hypertension with diabetes mellitus complications. Hypertens Res Off J Jpn Soc Hypertens. (2018) 41:147−56. doi: 10.1038/s41440-017-0008-y 29353881

[4] Henning RJ. Type-2 diabetes mellitus and cardiovascular disease. Future Cardiol. (2018) 14:491−509. doi: 10.2217/fca-2018-0045 30409037

[5] Palau V Riera M Soler MJ. The reno-cardiovascular connection in the patient with Diabetes mellitus: What’s new? Endocrinol Diabetes Nutr. (2017) 64:237−40. doi: 10.1016/j.endien.2017.03.011 28495318

[6] Sabanayagam C Banu R Chee ML Lee R Wang YX Tan G. Incidence and progression of diabetic retinopathy: a systematic review. Lancet Diabetes Endocrinol. (2019) 7:140−9. doi: 10.1016/S2213-8587(18)30128-1 30005958

[7] Zakin E Abrams R Simpson DM. Diabetic neuropathy. Semin Neurol. (2019) 39:560−9. doi: 10.1055/s-0039-1688978 31639839

[8] Zhang P Lu J Jing Y Tang S Zhu D Bi Y. Global epidemiology of diabetic foot ulceration: a systematic review and meta-analysis. Ann Med. (2017) 49:106−16. doi: 10.1080/07853890.2016.1231932 27585063

[9] Barnes JA Eid MA Creager MA Goodney PP. Epidemiology and risk of amputation in patients with diabetes mellitus and peripheral artery disease. Arterioscler Thromb Vasc Biol. (2020) 40:1808−17. doi: 10.1161/ATVBAHA.120.314595 32580632 PMC7377955

[10] Preshaw PM Alba AL Herrera D Jepsen S Konstantinidis A Makrilakis K. Periodontitis and diabetes: a two-way relationship. Diabetologia. (2012) 55:21−31. doi: 10.1007/s00125-011-2342-y 22057194 PMC3228943

[11] Navarro-Flores E Cauli O. Quality of life in individuals with diabetic foot syndrome. Endocr Metab Immune Disord Drug Targets. (2020) 20:1365−72. doi: 10.2174/1871530320666200128154036 32003676

[12] Simpson TC Clarkson JE Worthington HV MacDonald L Weldon JC Needleman I. Treatment of periodontitis for glycemic control in people with diabetes mellitus. Cochrane Database Syst Rev. (2022) 4:CD004714. doi: 10.1002/14651858.CD004714.pub4 35420698 PMC9009294

[13] Joseph JJ Deedwania P Acharya T Aguilar D Bhatt DL Chyun DA. Comprehensive management of cardiovascular risk factors for adults with type 2 diabetes: A scientific statement from the american heart association. Circulation. (2022) 145:e722−59. doi: 10.1161/CIR.0000000000001040 35000404

[14] Teo E Hassan N Tam W Koh S. Effectiveness of continuous glucose monitoring in maintaining glycemic control among people with type 1 diabetes mellitus: a systematic review of randomized controlled trials and meta-analysis. Diabetologia. (2022) 65:604−19. doi: 10.1007/s00125-021-05648-4 35141761

[15] Cortey A Renesme L Raignoux J Bedu A Casper C Tourneux P. Management of jaundice in the newborn≥35 GW: From screening to follow-up after discharge. Guidelines for clinical practice. Arch Pediatr Organe Off Soc Francaise Pediatr. (2017) 24:192−203. doi: 10.1016/j.arcped.2016.11.011 28094087

[16] Geoffroy PA Schroder CM Reynaud E Bourgin P. Efficacy of light therapy versus antidepressant drugs, and of the combination versus monotherapy, in major depressive episodes: A systematic review and meta-analysis. Sleep Med Rev déc. (2019) 48:101213. doi: 10.1016/j.smrv.2019.101213 31600678

[17] Mester E Szende B Spiry T Scher A. Stimulation of wound healing by laser rays. Acta Chir Acad Sci Hung. (1972) 13:315−24. 4659882

[18] Mester E Szende B Gärtner P. The effect of laser beams on the growth of hair in mice. Radiobiol Radiother (Berl). (1968) 9:621−6. 5732466

[19] Cotler HB. A NASA discovery has current applications in orthopedics. Curr Orthop Pract. (2015) 26:72. doi: 10.1097/BCO.0000000000000196 25541589 PMC4272231

[20] Heiskanen V Hamblin MR. Photobiomodulation: lasers vs. light emitting diodes? Photochem Photobiol Sci Off J Eur Photochem Assoc Eur Soc Photobiol. (2018) 17:1003−17. doi: 10.1039/c8pp00176f PMC6091542 30044464

[21] Glass GE. Photobiomodulation: A review of the molecular evidence for low level light therapy. J Plast Reconstr Aesthetic Surg JPRAS. (2021) 74:1050−60. doi: 10.1016/j.bjps.2020.12.059 33436333

[22] Desmet KD Paz DA Corry JJ Eells JT Wong-Riley MTT Henry MM. Clinical and experimental applications of NIR-LED photobiomodulation. Photomed Laser Surg. (2006) 24:121−8. doi: 10.1089/pho.2006.24.121 16706690

[23] Pastore D Greco M Passarella S. Specific helium-neon laser sensitivity of the purified cytochrome c oxidase. Int J Radiat Biol. (2000) 76:863−70. doi: 10.1080/09553000050029020 10902741

[24] Hamblin MR. Mechanisms and applications of the anti-inflammatory effects of photobiomodulation. AIMS Biophys. (2017) 4:337−61. doi: 10.3934/biophy.2017.3.337 28748217 PMC5523874

[25] Dompe C Moncrieff L Matys J Grzech-Leśniak K Kocherova I Bryja A. Photobiomodulation-underlying mechanism and clinical applications. J Clin Med. (2020) 9:1724. doi: 10.3390/jcm9061724 32503238 PMC7356229

[26] Zein R Selting W Hamblin MR. Review of light parameters and photobiomodulation efficacy: dive into complexity. J BioMed Opt. (2018) 23:1−17. doi: 10.1117/1.JBO.23.12.120901 PMC8355782 30550048

[27] Home - clinicalTrials.gov (2023). Available at: https://clinicaltrials.gov/.

[28] Harkless LB DeLellis S Carnegie DH Burke TJ. Improved foot sensitivity and pain reduction in patients with peripheral neuropathy after treatment with monochromatic infrared photo energy–MIRE. J Diabetes Complications. (2006) 20:81−7. doi: 10.1016/j.jdiacomp.2005.06.002 16504836

[29] DeLellis SL Carnegie DH Burke TJ. Improved sensitivity in patients with peripheral neuropathy: effects of monochromatic infrared photo energy. J Am Podiatr Med Assoc. (2005) 95:143−7. doi: 10.7547/0950143 15778471

[30] Kochman AB Carnegie DH Burke TJ. Symptomatic reversal of peripheral neuropathy in patients with diabetes. J Am Podiatr Med Assoc. (2002) 92:125−30. doi: 10.7547/87507315-92-3-125 11904323

[31] Anjo M Ummer VS Maiya AG Hande M B VS. Effect of photobiomodulation on serum neuron specific enolase (NSE) among patients with diabetic peripheral neuropathy - A pilot study. Diabetes Metab Syndr. (2020) 14:1061−3. doi: 10.1016/j.dsx.2020.06.065 32645648

[32] Anju M Chacko L Chettupalli Y Maiya AG Saleena Ummer V. Effect of Low Level Laser Therapy on serum vitamin D and magnesium levels in patients with diabetic peripheral neuropathy - A pilot study. Diabetes Metab Syndr. (2019) 13:1087−91. doi: 10.1016/j.dsx.2019.01.022 31336449

[33] Yamany AA Sayed HM. Effect of low level laser therapy on neurovascular function of diabetic peripheral neuropathy. J Adv Res. (2012) 3:21−8. doi: 10.1016/j.jare.2011.02.009

[34] Kamatham SA Chava VK. Comparison of salivary calprotectin levels in periodontitis associated with diabetes mellitus after low-level laser therapy as an adjunct to scaling and root planning: A randomized clinical trial. J Indian Soc Periodontol. (2022) 26:143−50. doi: 10.4103/jisp.jisp_149_21 35321295 PMC8936012

[35] Pulivarthi P Chava VK Gunupati S. Salivary tumor necrosis factor-alpha levels in periodontitis associated with diabetes mellitus after low level laser therapy as an adjunct to scaling and root planning: A randomized clinical trial. J Indian Soc Periodontol. (2022) 26:236−44. doi: 10.4103/jisp.jisp_150_21 35602530 PMC9118935

[36] Kocak E Sağlam M Arslan U Kayis SA Kebapcilar L Loos BG. Effect of diode laser application as an adjunct to nonsurgical periodontal therapy on the reduction of red complex microorganisms in type 2 diabetics with chronic periodontitis. Lasers Med Sci. (2020) 35:1403−10. doi: 10.1007/s10103-020-02997-1 32193820

[37] Koçak E Sağlam M Kayış SA Dündar N Kebapçılar L Loos BG. Nonsurgical periodontal therapy with/without diode laser modulates metabolic control of type 2 diabetics with periodontitis: a randomized clinical trial. Lasers Med Sci. (2016) 31:343−53. doi: 10.1007/s10103-016-1868-0 26754181

[38] Obradović R Kesić L Mihailović D Antić S Jovanović G Petrović A. A histological evaluation of a low-level laser therapy as an adjunct to periodontal therapy in patients with diabetes mellitus. Lasers Med Sci. (2013) 28:19−24. doi: 10.1007/s10103-012-1058-7 22311659

[39] Obradović R Kesić L Mihailović D Jovanović G Antić S Brkić Z. Low-level lasers as an adjunct in periodontal therapy in patients with diabetes mellitus. Diabetes Technol Ther. (2012) 14:799−803. doi: 10.1089/dia.2012.0027 22928615 PMC3429330

[40] Obradović R Kesić L Jovanović G Petrović D Goran R Mihailović D. Low power laser efficacy in the therapy of inflamed gingive in diabetics with parodontopathy. Vojnosanit Pregl. (2011) 68:684−9. doi: 10.2298/VSP1108684O 21991792

[41] de Oliveira AR da Silva FS Bortolin RH Marques DE da S Ramos GV Marqueti RC. Effect of photobiomodulation and exercise on early remodeling of the Achilles tendon in streptozotocin-induced diabetic rats. PloS One. (2019) 14:e0211643. doi: 10.1371/journal.pone.0211643 30716140 PMC6361457

[42] Linares SN Beltrame T Galdino GAM Frade MCM Milan-Mattos JC Gois MO. Dose response effect of photobiomodulation on hemodynamic responses and glucose levels in men with type 2 diabetes: A randomized, crossover, double-blind, sham-controlled trial. Photonics. juill. (2022) 9:481. doi: 10.3390/photonics9070481

[43] Milan-Mattos JC de Oliveira Francisco C Ferroli-Fabrício AM Minatel V Marcondes ACA Porta A. Acute effect of photobiomodulation using light-emitting diodes (LEDs) on baroreflex sensitivity during and after constant loading exercise in patients with type 2 diabetes mellitus. Lasers Med Sci. (2020) 35:329−36. doi: 10.1007/s10103-019-02815-3 31203569

[44] da Silva Leal MV Lima MO Nicolau RA de Carvallho TMT Abreu JA de C Pessoa DR. Effect of modified laser transcutaneous irradiation on pain and quality of life in patients with diabetic neuropathy. Photobiomodulation Photomed Laser Surg. (2020) 38:138−44. doi: 10.1089/photob.2019.4714 32195640

[45] de Alencar Fonseca Santos J Campelo MBD de Oliveira RA Nicolau RA Rezende VEA Arisawa EÂL. Effects of low-power light therapy on the tissue repair process of chronic wounds in diabetic feet. Photomed Laser Surg. (2018) 36:298−304. doi: 10.1089/pho.2018.4455 29882738

[46] Schindl A Heinze G Schindl M Pernerstorfer-Schön H Schindl L. Systemic effects of low-intensity laser irradiation on skin microcirculation in patients with diabetic microangiopathy. Microvasc Res. (2002) 64:240−6. doi: 10.1006/mvre.2002.2429 12204648

[47] Schindl M Kerschan K Schindl A Schön H Heinzl H Schindl L. Induction of complete wound healing in recalcitrant ulcers by low-intensity laser irradiation depends on ulcer cause and size. Photodermatol Photoimmunol Photomed. (1999) 15:18−21. doi: 10.1111/j.1600-0781.1999.tb00047.x 9990664

[48] Schindl A Schindl M Schön H Knobler R Havelec L Schindl L. Low-intensity laser irradiation improves skin circulation in patients with diabetic microangiopathy. Diabetes Care. (1998) 21:580−4. doi: 10.2337/diacare.21.4.580 9571346

[49] Vieira WF Malange KF de Magalhães SF Lemes JBP Dos Santos GG Nishijima CM. Anti-hyperalgesic effects of photobiomodulation therapy (904 nm) on streptozotocin-induced diabetic neuropathy imply MAPK pathway and calcium dynamics modulation. Sci Rep. (2022) 12:16730. doi: 10.1038/s41598-022-19947-2 36202956 PMC9537322

[50] Rocha IRC Perez-Reyes E Chacur M. Effect of photobiomodulation on mitochondrial dynamics in peripheral nervous system in streptozotocin-induced type 1 diabetes in rats. Photochem Photobiol Sci Off J Eur Photochem Assoc Eur Soc Photobiol. (2021) 20:293−301. doi: 10.1007/s43630-021-00018-w 33721255

[51] Vieira WF de Magalhães SF Farias FH de Thomaz AA Parada CA. Raman spectroscopy of dorsal root ganglia from streptozotocin-induced diabetic neuropathic rats submitted to photobiomodulation therapy. J Biophotonics. nov. (2019) 12:e201900135. doi: 10.1002/jbio.201900135 31265175

[52] Abdel-Wahhab KG Daoud EM El Gendy A Mourad HH Mannaa FA Saber MM. Efficiencies of low-level laser therapy (LLLT) and gabapentin in the management of peripheral neuropathy: diabetic neuropathy. Appl Biochem Biotechnol. (2018) 186:161−73. doi: 10.1007/s12010-018-2729-z 29527628

[53] da Silva Oliveira VR Cury DP Yamashita LB Esteca MV Watanabe IS Bergmann YF. Photobiomodulation induces antinociception, recovers structural aspects and regulates mitochondrial homeostasis in peripheral nerve of diabetic mice. J Biophotonics. (2018) 11:e201800110. doi: 10.1002/jbio.201800110 29749025

[54] Rastogi A Uppula P Saikia U Bhansali A. Effect of monochromatic infrared energy on quality of life and intraepidermal nerve fiber density in painful diabetic neuropathy: A randomized, sham control study. Neurol India. (2021) 69:1331−7. doi: 10.4103/0028-3886.329614 34747807

[55] Cg SK Maiya AG Hande HM Vidyasagar S Rao K Rajagopal KV. Efficacy of low level laser therapy on painful diabetic peripheral neuropathy. Laser Ther. (2015) 24:195−200. doi: 10.5978/islsm.15-OR-12 26557734 PMC4639677

[56] Bashiri H. Evaluation of low level laser therapy in reducing diabetic polyneuropathy related pain and sensorimotor disorders. Acta Med Iran. (2013) 51:543−7. 24026991

[57] Khamseh ME Kazemikho N Aghili R Forough B Lajevardi M Hashem Dabaghian F. Diabetic distal symmetric polyneuropathy: effect of low-intensity laser therapy. Lasers Med Sci. (2011) 26:831−5. doi: 10.1007/s10103-011-0977-z 21853320

[58] Swislocki A Orth M Bales M Weisshaupt J West C Edrington J. A randomized clinical trial of the effectiveness of photon stimulation on pain, sensation, and quality of life in patients with diabetic peripheral neuropathy. J Pain Symptom Manage. (2010) 39:88−99. doi: 10.1016/j.jpainsymman.2009.05.021 19896325

[59] Lavery LA Murdoch DP Williams J Lavery DC. Does anodyne light therapy improve peripheral neuropathy in diabetes? A double-blind, sham-controlled, randomized trial to evaluate monochromatic infrared photoenergy. Diabetes Care. (2008) 31:316−21. doi: 10.2337/dc07-1794 17977931

[60] Arnall DA Nelson AG López L Sanz N Iversen L Sanz I. The restorative effects of pulsed infrared light therapy on significant loss of peripheral protective sensation in patients with long-term type 1 and type 2 diabetes mellitus. Acta Diabetol. (2006) 43:26−33. doi: 10.1007/s00592-006-0207-5 16710647

[61] Clifft JK Kasser RJ Newton TS Bush AJ. The effect of monochromatic infrared energy on sensation in patients with diabetic peripheral neuropathy: a double-blind, placebo-controlled study. Diabetes Care. (2005) 28:2896−900. doi: 10.2337/diacare.28.12.2896 16306551

[62] Leonard DR Farooqi MH Myers S. Restoration of sensation, reduced pain, and improved balance in subjects with diabetic peripheral neuropathy: a double-blind, randomized, placebo-controlled study with monochromatic near-infrared treatment. Diabetes Care. (2004) 27:168−72. doi: 10.2337/diacare.27.1.168 14693984

[63] Zinman LH Ngo M Ng ET Nwe KT Gogov S Bril V. Low-intensity laser therapy for painful symptoms of diabetic sensorimotor polyneuropathy: a controlled trial. Diabetes Care. (2004) 27:921−4. doi: 10.2337/diacare.27.4.921 15047649

[64] Ahmed SA Ghoneim DF Morsy ME Hassan AA Mahmoud ARH. Low-level laser therapy with 670 nm alleviates diabetic retinopathy in an experimental model. J Curr Ophthalmol. (2021) 33:143−51. doi: 10.4103/JOCO.JOCO_29_20 34409224 PMC8365584

[65] Cheng Y Du Y Liu H Tang J Veenstra A Kern TS. Photobiomodulation inhibits long-term structural and functional lesions of diabetic retinopathy. Diabetes. (2018) 67:291−8. doi: 10.2337/db17-0803 29167189 PMC5780063

[66] Saliba A Du Y Liu H Patel S Roberts R Berkowitz BA. Photobiomodulation mitigates diabetes-induced retinopathy by direct and indirect mechanisms: evidence from intervention studies in pigmented mice. PloS One. (2015) 10:e0139003. doi: 10.1371/journal.pone.0139003 26426815 PMC4591336

[67] Tang J Du Y Lee CA Talahalli R Eells JT Kern TS. Low-intensity far-red light inhibits early lesions that contribute to diabetic retinopathy: *in vivo* and *in vitro* . Invest Ophthalmol Vis Sci. (2013) 54:3681−90. doi: 10.1167/iovs.12-11018 23557732 PMC3668802

[68] Shen W Teo KYC Wood JPM Vaze A Chidlow G Ao J. Preclinical and clinical studies of photobiomodulation therapy for macular oedema. Diabetologia. (2020) 63:1900−15. doi: 10.1007/s00125-020-05189-2 32661752

[69] Eells JT Gopalakrishnan S Connor TB Stepien K Carroll J Williams V. 670 nm photobiomodulation as a therapy for diabetic macular edema: A pilot study. Invest Ophthalmol Vis Sci. (2017) 58:932.

[70] Min SH Kwon J Do EJ Kim SH Kim ES Jeong JY. Duodenal dual-wavelength photobiomodulation improves hyperglycemia and hepatic parameters with alteration of gut microbiome in type 2 diabetes animal model. Cells. (2022) 11:3490. doi: 10.3390/cells11213490 36359885 PMC9654760

[71] Bonifacio M Benfato ID de Almeida Cruz M de Sales DC Pandolfo IL Quintana HT. Effects of photobiomodulation on glucose homeostasis and morphometric parameters in pancreatic islets of diabetic mice. Lasers Med Sci. (2022) 37:1799−809. doi: 10.1007/s10103-021-03434-7 34604943

[72] Gong L Zou Z Liu L Guo S Xing D. Photobiomodulation therapy ameliorates hyperglycemia and insulin resistance by activating cytochrome c oxidase-mediated protein kinase B in muscle. Aging. (2021) 13:10015−33. doi: 10.18632/aging.v13i7 33795530 PMC8064177

[73] Gong L Zou Z Huang L Guo S Xing D. Photobiomodulation therapy decreases free fatty acid generation and release in adipocytes to ameliorate insulin resistance in type 2 diabetes. Cell Signal. (2020) 67:109491. doi: 10.1016/j.cellsig.2019.109491 31809873

[74] Guo S Gong L Shen Q Xing D. Photobiomodulation reduces hepatic lipogenesis and enhances insulin sensitivity through activation of CaMKKβ/AMPK signaling pathway. J Photochem Photobiol B. (2020) 213:112075. doi: 10.1016/j.jphotobiol.2020.112075 33152638

[75] Hsu YH Chen YC Chen YW Chiu TH Kuo YT Chen CH. Far-infrared radiation prevents decline in β-cell mass and function in diabetic mice via the mitochondria-mediated Sirtuin1 pathway. Metabolism. (2020) 104:154143. doi: 10.1016/j.metabol.2020.154143 31927009

[76] Silva G Ferraresi C de Almeida RT Motta ML Paixão T Ottone VO. Insulin resistance is improved in high-fat fed mice by photobiomodulation therapy at 630 nm. J Biophotonics. mars. (2020) 13:e201960140. doi: 10.1002/jbio.201960140 31707768

[77] Silva G Ferraresi C de Almeida RT Motta ML Paixão T Ottone VO. Infrared photobiomodulation (PBM) therapy improves glucose metabolism and intracellular insulin pathway in adipose tissue of high-fat fed mice. Lasers Med Sci. (2018) 33:559−71. doi: 10.1007/s10103-017-2408-2 29247431

[78] Yoshimura TM Sabino CP Ribeiro MS. Photobiomodulation reduces abdominal adipose tissue inflammatory infiltrate of diet-induced obese and hyperglycemic mice. J Biophotonics. (2016) 9:1255−62. doi: 10.1002/jbio.201600088 27635634

[79] Scontri CMCB de Castro Magalhães F Damiani APM Hamblin MR Zamunér AR Ferraresi C. Dose and time-response effect of photobiomodulation therapy on glycemic control in type 2 diabetic patients combined or not with hypoglycemic medicine: a randomized, crossover, double-blind, sham controlled trial. J Biophotonics. (2023) 16:e202300083. doi: 10.1002/jbio.202300083 37171054 PMC10662441

[80] da Silva Tonetto L da Silva CCF Gonzatti N Guex CG Hartmann DD Boschi ES. Effects of photobiomodulation on oxidative stress in rats with type 2 diabetes mellitus. Lasers Med Sci. (2023) 38:90. doi: 10.1007/s10103-023-03745-x 36947266

[81] Frigero M Dos Santos SA Serra AJ Dos Santos Monteiro MaChado C Portes LA Tucci PJF. Effect of photobiomodulation therapy on oxidative stress markers of gastrocnemius muscle of diabetic rats subjected to high-intensity exercise. Lasers Med Sci. (2018) 33:1781−90. doi: 10.1007/s10103-018-2540-7 29808322

[82] Gobbi A de Carvalho G Sapalo AT de Jesus Guirro RR. Acute application of photobiomodulation does not bring important gains for the muscular performance and functionality of diabetic individuals. Lasers Med Sci. (2021) 36:995−1002. doi: 10.1007/s10103-020-03135-7 32862403

[83] Francisco C de O Beltrame T Hughson RL Milan-Mattos JC Ferroli-Fabricio AM Galvão Benze B. Effects of light-emitting diode therapy (LEDT) on cardiopulmonary and hemodynamic adjustments during aerobic exercise and glucose levels in patients with diabetes mellitus: A randomized, crossover, double-blind and placebo-controlled clinical trial. Complement Ther Med. (2019) 42:178−83. doi: 10.1016/j.ctim.2018.11.015 30670240

[84] Dungel P Sutalo S Slezak C Keibl C Schädl B Schnidar H. Wavelength-dependent effects of photobiomodulation for wound care in diabetic wounds. Int J Mol Sci. (2023) 24:5895. doi: 10.3390/ijms24065895 36982967 PMC10054229

[85] Ebrahimpour-Malekshah R Amini A Mostafavinia A Ahmadi H Zare F Safaju S. The stereological, immunohistological, and gene expression studies in an infected ischemic wound in diabetic rats treated by human adipose-derived stem cells and photobiomodulation. Arch Dermatol Res. (2023) 1717–34. doi: 10.1007/s00403-023-02563-z 36808225

[86] Mehrvar S Mostaghimi S Foomani FH Abroe B Eells JT Gopalakrishnan S. 670 nm photobiomodulation improves the mitochondrial redox state of diabetic wounds. Quant Imaging Med Surg. (2021) 11:107−18. doi: 10.21037/qims 33392015 PMC7719930

[87] Ahmadi H Amini A Fadaei Fathabady F Mostafavinia A Zare F Ebrahimpour-Malekshah R. Transplantation of photobiomodulation-preconditioned diabetic stem cells accelerates ischemic wound healing in diabetic rats. Stem Cell Res Ther. (2020) 11:494. doi: 10.1186/s13287-020-01967-2 33239072 PMC7688005

[88] Bagheri M Mostafavinia A Abdollahifar MA Amini A Ghoreishi SK Chien S. Combined effects of metformin and photobiomodulation improve the proliferation phase of wound healing in type 2 diabetic rats. BioMed Pharmacother Biomedecine Pharmacother. (2020) 123:109776. doi: 10.1016/j.biopha.2019.109776 31911295

[89] Kouhkheil R Fridoni M Abdollhifar MA Amini A Bayat S Ghoreishi SK. Impact of photobiomodulation and condition medium on mast cell counts, degranulation, and wound strength in infected skin wound healing of diabetic rats. Photobiomodulation Photomed Laser Surg. (2019) 37:706−14. doi: 10.1089/photob.2019.4691 31589095

[90] Fekrazad R Sarrafzadeh A Kalhori KAM Khan I Arany PR Giubellino A. Improved wound remodeling correlates with modulated TGF-beta expression in skin diabetic wounds following combined red and infrared photobiomodulation treatments. Photochem Photobiol. (2018) 94:775−9. doi: 10.1111/php.12914 29457837

[91] Asghari M Kanonisabet A Safakhah M Azimzadeh Z Mostafavinia A Taheri S. The effect of combined photobiomodulation and metformin on open skin wound healing in a non-genetic model of type II diabetes. J Photochem Photobiol B. (2017) 169:63−9. doi: 10.1016/j.jphotobiol.2017.03.002 28282557

[92] Leite G de PMF das Neves LMS Silva CA Guirro RR de J de Souza TR de Souza AK. Photobiomodulation laser and pulsed electrical field increase the viability of the musculocutaneous flap in diabetic rats. Lasers Med Sci. (2017) 32:641−8. doi: 10.1007/s10103-017-2160-7 28155011

[93] Fahimipour F Houshmand B Alemi P Asnaashari M Tafti MA Akhoundikharanagh F. The effect of He-Ne and Ga-Al-As lasers on the healing of oral mucosa in diabetic mice. J Photochem Photobiol B. (2016) 159:149−54. doi: 10.1016/j.jphotobiol.2016.03.020 27062456

[94] Fekrazad R Mirmoezzi A Kalhori KA Arany P. The effect of red, green and blue lasers on healing of oral wounds in diabetic rats. J Photochem Photobiol B. (2015) 148:242−5. doi: 10.1016/j.jphotobiol.2015.04.018 25981185

[95] Dancáková L Vasilenko T Kováč I Jakubčová K Hollý M Revajová V. Low-level laser therapy with 810 nm wavelength improves skin wound healing in rats with streptozotocin-induced diabetes. Photomed Laser Surg. (2014) 32:198−204. doi: 10.1089/pho.2013.3586 24661084 PMC3985531

[96] Aparecida Da Silva A Leal-Junior ECP Alves ACA Rambo CS Dos Santos SA Vieira RP. Wound-healing effects of low-level laser therapy in diabetic rats involve the modulation of MMP-2 and MMP-9 and the redistribution of collagen types I and III. J Cosmet Laser Ther Off Publ Eur Soc Laser Dermatol. (2013) 15:210−6. doi: 10.3109/14764172.2012.761345 23463906

[97] Fathabadie FF Bayat M Amini A Bayat M Rezaie F. Effects of pulsed infra-red low level-laser irradiation on mast cells number and degranulation in open skin wound healing of healthy and streptozotocin-induced diabetic rats. J Cosmet Laser Ther Off Publ Eur Soc Laser Dermatol. (2013) 15:294−304. doi: 10.3109/14764172.2013.764435 23463989

[98] Firat ET Dağ A Günay A Kaya B Karadede Mİ Kanay BE. The effects of low-level laser therapy on palatal mucoperiosteal wound healing and oxidative stress status in experimental diabetic rats. Photomed Laser Surg. (2013) 31:315−21. doi: 10.1089/pho.2012.3406 23789588

[99] Dadpay M Sharifian Z Bayat M Bayat M Dabbagh A. Effects of pulsed infra-red low level-laser irradiation on open skin wound healing of healthy and streptozotocin-induced diabetic rats by biomechanical evaluation. J Photochem Photobiol B. (2012) 111:1−8. doi: 10.1016/j.jphotobiol.2012.03.001 22494918

[100] Park JJ Kang KL. Effect of 980-nm GaAlAs diode laser irradiation on healing of extraction sockets in streptozotocin-induced diabetic rats: a pilot study. Lasers Med Sci. (2012) 27:223−30. doi: 10.1007/s10103-011-0944-8 21732114

[101] Hegde VN Prabhu V Rao SBS Chandra S Kumar P Satyamoorthy K. Effect of laser dose and treatment schedule on excision wound healing in diabetic mice. Photochem Photobiol. (2011) 87:1433−41. doi: 10.1111/j.1751-1097.2011.00991.x 21883243

[102] Peplow PV Chung TY Ryan B Baxter GD. Laser photobiostimulation of wound healing: reciprocity of irradiance and exposure time on energy density for splinted wounds in diabetic mice. Lasers Surg Med. (2011) 43:843−50. doi: 10.1002/lsm.21094 21956633

[103] Carvalho PTCde Silva IS Reis FA Perreira DM Aydos RD. Influence of ingaalp laser (660nm) on the healing of skin wounds in diabetic rats. Acta Cir Bras. (2010) 25:71−9. doi: 10.1590/S0102-86502010000100016 20126892

[104] Akyol U Güngörmüş M. The effect of low-level laser therapy on healing of skin incisions made using a diode laser in diabetic rats. Photomed Laser Surg. (2010) 28:51−5. doi: 10.1089/pho.2008.2425 19754259

[105] Chung TY Peplow PV Baxter GD. Laser photobiostimulation of wound healing: defining a dose response for splinted wounds in diabetic mice. Lasers Surg Med. (2010) 42:656−64. doi: 10.1002/lsm.20981 20976805

[106] Santos NRS dos Santos JN dos Reis JA Oliveira PC de Sousa APC de Carvalho CM. Influence of the use of laser phototherapy (lambda660 or 790 nm) on the survival of cutaneous flaps on diabetic rats. Photomed Laser Surg. (2010) 28:483−8. doi: 10.1089/pho.2009.2500 19831497

[107] Al-Watban FAH. Laser therapy converts diabetic wound healing to normal healing. Photomed Laser Surg. (2009) 27:127−35. doi: 10.1089/pho.2008.2406 19193104

[108] Güngörmüş M Akyol UK. Effect of biostimulation on wound healing in diabetic rats. Photomed Laser Surg. (2009) 27:607−10. doi: 10.1089/pho.2008.2349 19694508

[109] Maiya AG Kumar P Nayak S. Photo-stimulatory effect of low energy helium-neon laser irradiation on excisional diabetic wound healing dynamics in Wistar rats. Indian J Dermatol. (2009) 54:323−9. doi: 10.4103/0019-5154.57606 20101331 PMC2807706

[110] Carvalho PdeTCde Mazzer N dos Reis FA Belchior ACG Silva IS. Analysis of the influence of low-power HeNe laser on the healing of skin wounds in diabetic and non-diabetic rats. Acta Cir Bras. (2006) 21:177−83. doi: 10.1590/S0102-86502006000300010 16751932

[111] Rabelo SB Villaverde AB Nicolau R Salgado MC Melo MDS Pacheco MTT. Comparison between wound healing in induced diabetic and nondiabetic rats after low-level laser therapy. Photomed Laser Surg. (2006) 24:474−9. doi: 10.1089/pho.2006.24.474 16942427

[112] Maiya GA Kumar P Rao L. Effect of low intensity helium-neon (He-Ne) laser irradiation on diabetic wound healing dynamics. Photomed Laser Surg. (2005) 23:187−90. doi: 10.1089/pho.2005.23.187 15910184

[113] Byrnes KR Barna L Chenault VM Waynant RW Ilev IK Longo L. Photobiomodulation improves cutaneous wound healing in an animal model of type II diabetes. Photomed Laser Surg. (2004) 22:281−90. doi: 10.1089/1549541041797977 15345169

[114] Reddy GK Stehno-Bittel L Enwemeka CS. Laser photostimulation accelerates wound healing in diabetic rats. Wound Repair Regener Off Publ Wound Heal Soc Eur Tissue Repair Soc. (2001) 9:248−55. doi: 10.1046/j.1524-475x.2001.00248.x 11472621

[115] Haze A Gavish L Elishoov O Shorka D Tsohar T Gellman YN. Treatment of diabetic foot ulcers in a frail population with severe co-morbidities using at-home photobiomodulation laser therapy: a double-blind, randomized, sham-controlled pilot clinical study. Lasers Med Sci. (2022) 37:919−28. doi: 10.1007/s10103-021-03335-9 34052927

[116] Vitoriano NAM Mont’Alverne DGB Martins MIS Silva PS Martins CA Teixeira HD. Comparative study on laser and LED influence on tissue repair and improvement of neuropathic symptoms during the treatment of diabetic ulcers. Lasers Med Sci. (2019) 34:1365−71. doi: 10.1007/s10103-019-02724-5 30715637

[117] Frangež I Nizič-Kos T Frangež HB. Phototherapy with LED shows promising results in healing chronic wounds in diabetes mellitus patients: A prospective randomized double-blind study. Photomed Laser Surg. (2018) 36:377−82. doi: 10.1089/pho.2017.4382 29668397

[118] Ruh AC Frigo L Cavalcanti MFXB Svidnicki P Vicari VN Lopes-Martins RAB. Laser photobiomodulation in pressure ulcer healing of human diabetic patients: gene expression analysis of inflammatory biochemical markers. Lasers Med Sci. (2018) 33:165−71. doi: 10.1007/s10103-017-2384-6 29181642

[119] Mathur RK Sahu K Saraf S Patheja P Khan F Gupta PK. Low-level laser therapy as an adjunct to conventional therapy in the treatment of diabetic foot ulcers. Lasers Med Sci. (2017) 32:275−82. doi: 10.1007/s10103-016-2109-2 27896528

[120] Carvalho AFMde Feitosa MCP Coelho NPM de F Rebêlo VCN Castro JGde Sousa PRGde. Low-level laser therapy and Calendula officinalis in repairing diabetic foot ulcers. Rev Esc Enferm U P. (2016) 50:628−34. doi: 10.1590/S0080-623420160000500013 27680049

[121] Sandoval Ortíz MC Herrera Villabona E Camargo Lemos DM Castellanos R. Effects of low level laser therapy and high voltage stimulation on diabetic wound healing. Rev Univ Ind Santander Salud. (2014) 46:107−17.

[122] Kajagar BM Godhi AS Pandit A Khatri S. Efficacy of low level laser therapy on wound healing in patients with chronic diabetic foot ulcers-a randomized control trial. Indian J Surg. (2012) 74:359−63. doi: 10.1007/s12262-011-0393-4 24082586 PMC3477409

[123] Kaviani A Djavid GE Ataie-Fashtami L Fateh M Ghodsi M Salami M. A randomized clinical trial on the effect of low-level laser therapy on chronic diabetic foot wound healing: a preliminary report. Photomed Laser Surg. (2011) 29:109−14. doi: 10.1089/pho.2009.2680 21214368

[124] Minatel DG Frade MAC França SC Enwemeka CS. Phototherapy promotes healing of chronic diabetic leg ulcers that failed to respond to other therapies. Lasers Surg Med. (2009) 41:433−41. doi: 10.1002/lsm.20789 19588536

[125] Dalirsani Z Ghazi N Delavarian Z Pakfetrat A Esmaily H Davaji M. Effects of diode low-level laser therapy on healing of tooth extraction sockets: a histopathological study in diabetic rats. Lasers Med Sci. (2021) 36:1527−34. doi: 10.1007/s10103-021-03270-9 33644838

[126] Lee JH Kong SC Chen CH Lin YC Lee KT Wang YH. The effects of photobiomodulation on bone defect repairing in a diabetic rat model. Int J Mol Sci. (2021) 22:11026. doi: 10.3390/ijms222011026 34681687 PMC8541159

[127] Diker N Aytac D Helvacioglu F Dagdelen C Oguz Y. Evaluation of the effects of low-level laser therapy on diabetic bone healing. J Craniofac Surg. (2019) 30:1994−8. doi: 10.1097/SCS.0000000000005654 31232987

[128] Gomes MF Goulart M da GV Giannasi LC Hiraoka CM Melo G de FS Zangaro RA. Effects of the photobiomodulation using different energy densities on the periodontal tissues under orthodontic force in rats with type 2 diabetes mellitus. Braz Oral Res. (2018) 32:e61. doi: 10.1590/1807-3107bor-2018.vol32.0061 30379208

[129] Mostafavinia A Masteri Farahani R Abdollahifar MA Ghatrehsamani M Ghoreishi SK Hajihossainlou B. Evaluation of the effects of photobiomodulation on partial osteotomy in streptozotocin-induced diabetes in rats. Photomed Laser Surg. (2018) 36:406−14. doi: 10.1089/pho.2018.4438 29851368

[130] Mostafavinia A Razavi S Abdollahifar M Amini A Ghorishi SK Rezaei F. Evaluation of the effects of photobiomodulation on bone healing in healthy and streptozotocin-induced diabetes in rats. Photomed Laser Surg. (2017) 35:537−45. doi: 10.1089/pho.2016.4224 28358661

[131] Yildirimturk S Sirin Y Soluk Tekkesin M Gurler G Firat D. The effects of low-level laser therapy on the healing of bone defects in streptozotocin-induced diabetic rats: A histological and morphometric evaluation. J Cosmet Laser Ther Off Publ Eur Soc Laser Dermatol. (2017) 19:397−403. doi: 10.1080/14764172.2017.1341048 28622041

[132] Patrocínio-Silva TL Souza AMFde Goulart RL Pegorari CF Oliveira JR Fernandes KR. Low-level laser therapy associated to a resistance training protocol on bone tissue in diabetic rats. Arch Endocrinol Metab. Arch Endocrinol Metab. (2016) 60:457−64. doi: 10.1590/2359-3997000000190 27812609 PMC10118645

[133] Magri AMP Fernandes KR Assis L Mendes NA da Silva Santos ALY de Oliveira Dantas E. Photobiomodulation and bone healing in diabetic rats: evaluation of bone response using a tibial defect experimental model. Lasers Med Sci. (2015) 30:1949−57. doi: 10.1007/s10103-015-1789-3 26223384

[134] Nascimento MFdo Almeida BMde Cunha JLS Valois RBV Pinheiro JC Ribeiro MAG. Improvement of bone repair in diabetic rats subjected to ƛ780 nm low-level laser therapy. Acta Cir Bras. (2015) 30:660−7. doi: 10.1590/S0102-865020150100000002 26560423

[135] Patrocínio-Silva TL de Souza AMF Goulart RL Pegorari CF Oliveira JR Fernandes K. The effects of low-level laser irradiation on bone tissue in diabetic rats. Lasers Med Sci. (2014) 29:1357−64. doi: 10.1007/s10103-013-1418-y 23990218

[136] Akyol UK Güngörmüş M. Effect of biostimulation on healing of bone defects in diabetic rats. Photomed Laser Surg. (2010), 28 411−6. doi: 10.1089/pho.2008.2478 19860570

[137] Abdi S Bayat M Javadieh F Mohsenifar Z Rezaie F Bayat M. The effects of helium-neon light therapy on healing of partial osteotomy of the tibia in streptozotocin induced diabetic rats. Photomed Laser Surg. (2009) 27:907−12. doi: 10.1089/pho.2008.2421 20035603

[138] Bayat M Abdi S Javadieh F Mohsenifar Z Rashid MR. The effects of low-level laser therapy on bone in diabetic and nondiabetic rats. Photomed Laser Surg. (2009) 27:703−8. doi: 10.1089/pho.2008.2351 19698018

[139] Javadieh F Bayat M Abdi S Mohsenifar Z Razi S. The effects of infrared low-level laser therapy on healing of partial osteotomy of tibia in streptozotocin-induced diabetic rats. Photomed Laser Surg. (2009) 27:641−6. doi: 10.1089/pho.2008.2370 19694509

[140] Attia MS Elewa GM Abdelgawad N Ismail RM Hassan Eid M Ghoneim MM. The influence of low-level laser therapy on CBCT radiographic and biochemical profiles of type II controlled diabetic patients after dental implant insertion: A randomized case-control study. Cureus. mars. (2023) 15:e36559. doi: 10.7759/cureus.36559 PMC10123229 37102010

[141] Mrasori S Popovska M Rusevska B Shkreta M Selani A Bunjaku V. Effects of low level laser therapy (LLLT) on serum values of interleukin 6 (IL-6) in patients with periodontitis and type 2 diabetes mellitus (T2DM). Acta Inform Med AIM J Soc Med Inform Bosnia Herzeg Cas Drustva Za Med Inform BiH. (2021) 29:59−64. doi: 10.5455/aim. PMC8116077 34012215

[142] Soi S Bains VK Srivastava R Madan R. Comparative evaluation of improvement in periodontal and glycemic health status of type 2 diabetes mellitus patients after scaling and root planing with or without adjunctive use of diode laser. Lasers Med Sci. (2021) 36:1307−15. doi: 10.1007/s10103-021-03261-w 33521870

[143] Özberk SS Gündoğar H Özkaya M Taner İL Erciyas K. The effect of photobiomodulation therapy on nonsurgical periodontal treatment in patients with type 2 diabetes mellitus: a randomized controlled, single-blind, split-mouth clinical trial. Lasers Med Sci. (2020) 35:497−504. doi: 10.1007/s10103-019-02897-z 31641967

[144] Castro Dos Santos N Andere NMRB Miguel MMV Dos Santos LM Santamaria M Mathias IF. Photobiomodulation for the treatment of periodontal pockets in patients with type 2 diabetes: 1-year results of a randomized clinical trial. Lasers Med Sci. (2019) 34:1897−904. doi: 10.1007/s10103-019-02799-0 31093797

[145] Chandra S Shashikumar P. Diode laser - A novel therapeutic approach in the treatment of chronic periodontitis in type 2 diabetes mellitus patients: A prospective randomized controlled clinical trial. J Lasers Med Sci. (2019) 10:56−63. doi: 10.15171/jlms.2019.09 31360370 PMC6499582

[146] Dengizek Eltas S Gursel M Eltas A Alptekin NO Ataoglu T. Evaluation of long-term effects of diode laser application in periodontal treatment of poorly controlled type 2 diabetic patients with chronic periodontitis. Int J Dent Hyg. (2019) 17:292−9. doi: 10.1111/idh.12384 30697968

[147] Li F Xu HS. Effects of low level laser combined with basic periodontal therapy on cytokines and LPS, leptin in gingival crevicular fluid of diabetes mellitus complicated with chronic periodontitis patients. Shanghai Kou Qiang Yi Xue Shanghai J Stomatol. (2018) 27:637−40. 30899947

[148] Demirturk-Gocgun O Baser U Aykol-Sahin G Dinccag N Issever H Yalcin F. Role of low-level laser therapy as an adjunct to initial periodontal treatment in type 2 diabetic patients: A split-mouth, randomized, controlled clinical trial. Photomed Laser Surg. (2017) 35:111−5. doi: 10.1089/pho.2016.4117 27855270

[149] Javed F Al Amri MD Al-Kheraif AA Qadri T Ahmed A Ghanem A. Efficacy of non-surgical periodontal therapy with adjunct Nd : YAG laser therapy in the treatment of periodontal inflammation among patients with and without type 2 diabetes mellitus: A short-term pilot study. J Photochem Photobiol B. (2015) 149:230−4. doi: 10.1016/j.jphotobiol.2015.06.013 26103088

[150] Loe H Silness J. Periodontal disease in pregnancy. i. prevalence and severity. Acta Odontol Scand. (1963) 21:533−51. 14121956 10.3109/00016356309011240

[151] Silness J Loe H. Periodontal disease in pregnancy. ii. correlation between oral hygiene and periodontal condtion. Acta Odontol Scand. (1964) 22:121−35. 14158464 10.3109/00016356408993968

[152] Yang L Liu G Jiang D Lin G Ren Z Fan H. Effect of near-infrared laser treatment on improving erectile function in rats with diabetes mellitus. Andrology. (2023) 1472–83. doi: 10.1111/andr.13422 36869699

[153] Asghari A Takhtfooladi MA Hoseinzadeh HA. Effect of photobiomodulation on ischemia/reperfusion-induced renal damage in diabetic rats. Lasers Med Sci. (2016) 31:1943−8. doi: 10.1007/s10103-016-2073-x 27624783

[154] Aghamohamdi D Fakhari S Farhoudi M Farzin H. The efficacy of low-level laser therapy in the treatment of bell’s palsy in diabetic patients. J Lasers Med Sci. (2020) 11:310−5. doi: 10.34172/jlms.2020.52 32802293 PMC7369545

[155] Hode L. The importance of the coherency. Photomed Laser Surg. (2005) 23:431−4. doi: 10.1089/pho.2005.23.431 16144489

[156] Zalevsky Z Belkin M. Coherence and speckle in photomedicine and photobiology. Photomed Laser Surg. (2011) 29:655−6. doi: 10.1089/pho.2010.2939 21749264

[157] de Freitas LF Hamblin MR. Proposed mechanisms of photobiomodulation or low-level light therapy. IEEE J Sel Top Quantum Electron Publ IEEE Lasers Electro-Opt Soc. (2016) 22:7000417. doi: 10.1109/JSTQE.2016.2561201 PMC5215870 28070154

[158] Deana NF Zaror C Del Sol M Bagnato VS Alves N. Wound contraction rate in excised and unexcised burn wounds with laser photobiomodulation: Systematic review and meta-analysis of preclinical studies. Burns J Int Soc Burn Inj. (2023) 49:261−74. doi: 10.1016/j.burns.2022.05.009 35842272

[159] Lopes C de CA Limirio JPJO Zanatta LSA Simamoto VRN Dechichi P Limirio APHJO. Effectiveness of photobiomodulation therapy on human bone healing in dentistry: A systematic review. Photobiomodulation photomed laser surg. Photobiomodul Photomed Laser Surg. (2022) 40:440−53. doi: 10.1089/photob.2021.0092 35527692

[160] Ebrahimi P Hadilou M Naserneysari F Dolatabadi A Tarzemany R Vahed N. Effect of photobiomodulation in secondary intention gingival wound healing-a systematic review and meta-analysis. BMC Oral Health. (2021) 21:258. doi: 10.1186/s12903-021-01611-2 33985492 PMC8120828

[161] Ribu L Birkeland K Hanestad BR Moum T Rustoen T. A longitudinal study of patients with diabetes and foot ulcers and their health-related quality of life: wound healing and quality-of-life changes. J Diabetes Complications. (2008) 22:400−7. doi: 10.1016/j.jdiacomp.2007.06.006 18413188

[162] Ayuk SM Houreld NN Abrahamse H. Effect of 660 nm visible red light on cell proliferation and viability in diabetic models *in vitro* under stressed conditions. Lasers Med Sci. (2018) 33:1085−93. doi: 10.1007/s10103-017-2432-2 29520687

[163] De Marchi T Ferlito JV Ferlito MV Salvador M Leal-Junior ECP. Can photobiomodulation therapy (PBMT) minimize exercise-induced oxidative stress? A systematic review and meta-analysis. Antioxid Basel Switz. 27 août. (2022) 11:1671. doi: 10.3390/antiox11091671 PMC9495825 36139746

[164] Ferraresi C. Use of photobiomodulation therapy in exercise performance enhancement and postexercise recovery: true or myth? Photobiomodulation Photomed Laser Surg. (2020) 38:705−7. doi: 10.1089/photob.2020.4948 33216681

[165] Liebman C Loya S Lawrence M Bashoo N Cho M. Stimulatory responses in α- and β-cells by near-infrared (810 nm) photobiomodulation. J Biophotonics. (2022) 15:e202100257. doi: 10.1002/jbio.202100257 34837336

[166] Irani S Mohseni Salehi Monfared SS Akbari-Kamrani M Ostad SN Abdollahi M Larijani B. Effect of low-level laser irradiation on *in vitro* function of pancreatic islets. Transplant Proc. (2009) 41:4313−5. doi: 10.1016/j.transproceed.2009.09.065 20005390

[167] Huang HH Stillman TJ Branham LA Williams SC. The effects of photobiomodulation therapy on porcine islet insulin secretion. Photobiomodulation Photomed Laser Surg. (2022) 40:395−401. doi: 10.1089/photob.2022.0022 35594334

